# Effective removal of acetamiprid and eosin Y by adsorption on pristine and modified MIL-101(Fe)

**DOI:** 10.1007/s11356-024-33821-w

**Published:** 2024-06-07

**Authors:** Mohamed Sakr, Mina Shawky Adly, Mohamed Gar Alalm, Hani Mahanna

**Affiliations:** 1 Public Works Engineering Department, Faculty of Engineering, Mansoura University, Mansoura, 35516 Egypt; 2 Chemistry Department, Faculty of Science, Mansoura University, Mansoura, 35516 Egypt

**Keywords:** Adsorption, Acetamiprid, Eosin Y dye, Isotherm models, NH_2_-MIL-101(Fe), Wastewater

## Abstract

In this work, the efficacy of two metal–organic frameworks (MIL-101(Fe) and NH_2_-MIL-101(Fe)) in eliminating acetamiprid (ATP) insecticide and eosin Y (EY) dye from aqueous solution is tested. An analysis was conducted on the developed nanocomposite’s optical, morphological, and structural characteristics. The adsorption isotherm, kinetics, thermodynamics, reusability, and mechanisms for ATP and EY dye removal were assessed. NH_2_-MIL-101(Fe) adsorbed 76% and 90% of ATP pesticide and EY dye, respectively after 10 to 15 min in optimum conditions. For both adsorbents, with regard to explaining the isotherm data, the Langmuir model offered the most accurate description. Moreover, the adsorption of ATP and EY dye is described by the pseudo-second-order kinetic model. The maximum adsorption capacities of ATP and EY dye on MIL-101(Fe) were 57.6 and 48.9 mg/g compared to 70.5 and 97.8 mg/g using NH_2_-MIL-101(Fe). The greatest amount of ATP and EY dye clearance was obtained at a neutral medium for both adsorbents. The results of this investigation demonstrate the effectiveness of MIL-101(Fe) and NH_2_-MIL-101(Fe) as effective substances in the adsorption process for removing pesticides and dyes from aqueous solution.

**Supplementary Information:**

The online version contains supplementary material available at 10.1007/s11356-024-33821-w.

## Introduction

Water pollution is still a problem, and the security of water resources has a significant impact on human life (Zhang et al. 2022). A rise in emerging pollutants in the environment is being caused by anthropogenic activities and industrial outputs (Marican & Duran-Lara 2018). Due to the risks it poses to the environment and public health, water contamination by organic and inorganic substances is a pressing issue that has attracted attention worldwide (Doan et al. 2021). Because of the pollutants’ toxicity, persistence, and concentration, there are negative effects on the environment, public health, and the economy (Fua et al. 2014). Aside from other demands, there has been a significant growth in the water demand, with the agricultural, industrial, and home sectors absorbing, respectively, 70%, 22%, and 8% of the freshwater supply. Approximately 500 million tonnes of these microcontaminants are produced annually on a worldwide scale (Saleh et al. 2020). Large volumes of wastewater containing a variety of contaminants have been produced as a result of this. Wastewater must thus be treated prior to release into the environment (Zainorizuan et al. 2017). Due to their bio-accumulative property, which has a harmful influence on the environment and human health, pesticides of various types are a threat to the cosmos. Generally, hazardous chemical identification is a time-consuming and expensive operation on the other hand. Pesticide treatment from water faces many difficulties, including the impactful composition, the variety of pesticide physical structures, and the pH of the pesticide-tainted water. The variety of amounts of pesticides in various water sources is 0.1 to 107 mg. L^−1^. However, the effluent from the manufacture of pesticides needs to be thoroughly treated before it is mixed with domestic wastewater. Consequently, given their high concentrations and recalcitrance in wastewater and water sources treated with pesticides is a crucial scientific study (Anand et al. 2020; Saleh et al. 2020). Nowadays, neonicotinoid insecticides have been demonstrated to be a novel group of active components. Acetamiprid, clothianidin, thiamethoxam, and imidacloprid are the most widely used neonicotinoid insecticides (Ghiasi et al. 2022). Acetamiprid is a relatively new and effective neonicotinoid pesticide and traces of it have a critical impact on water (2.50 ng. L^−1^, 0.2–7.7 μg. L^−1^) (Zoumenou et al. 2019). In rats, oral administration of 217 mg.kg^−1^ is the average fatal dosage. The buildup of this pesticide in different tissues is tightly linked to both direct and indirect exposure to it (Pisa et al. 2021). Research has shown that mice’s liver, kidney, and other tissues contain notable amounts of acetamiprid and its metabolites (Ford & Casida 2006). According to several research on acetamiprid exposure, oxidative stress production might be the overall mechanism behind the toxicity of pesticides to several organs in animal studies. Mammals exposed to acetamiprid have shown signs of oxidative stress, ptosis, and DNA damage (Annabi et al. 2019, Ayyavoo & Tamilselvan 2019).

The global manufacturing of synthetic dyes is estimated by tons of the total pigments produced annually, and about 10–60% of the effluent containing the dye which emitted into the environment without proper treatment. Dye metabolites that have been subjected to oxidation, heat, and light are very hazardous and have cancer-causing properties (Assefi et al. 2014, Borzelleca et al. 1987). When trying to remove it, we may face many difficulties including dye decomposition since these dyes are not biodegradable and have a high resilience against oxidation and light (Mahanna & El-Bendary 2022).

2-(2,4,5,6- Tetrabromo-6-oxido-3-oxido-3H xanthenes-9-yl) benzoate disodium salt is the IUPAC name for eosin yellow, an anionic dye (Ansari et al. 2016). It belongs to the fluorescein dye family and is very water-soluble. Eosin Yellow is frequently used in gram staining to distinguish between various bacterial species because of its remarkable capacity to absorb red blood cells and red color (Oladipo et al. 2021). EY dye is a unique acid dye often used for printing, tanning, and coloring leather, cotton, printing, and other materials (Jabbar et al. 2022) (Singh et al. 2017). According to Eosin Yellow’s toxicological data, the EY dye could seriously irritate skin and eyes. When the dye physically touches the skin, it irritates it, resulting in discomfort, redness, and swelling. When consumed, it has several negative consequences, especially on the body's essential organs including the liver and kidney. Direct dye contact with the eye can permanently damage the cornea. The DNA in living things’ gastrointestinal organs is also damaged, which leads to an assortment of ailments within a human being. The ability of the lungs to exchange gases is decreased by dye inhalation. Additionally, dyes can be poisonous and carcenogenic, metabolites (Sharma et al. 2019; Silva et al. 2018, Thabet & Ismaiel 2016).

Coordination polymers/networks or metal–organic frameworks (MOFs) are a novel category of hybrid porous crystalline materials that emerge from the interaction of multidentate organic linkers with inorganic vertices, such as metal ions or clusters, acting as nodes (Trung et al. 2011). MOFs are characterized by microporous structures that hold larger pore sizes called MIL (Material of Institute Lavoisier) (Rasheed et al. 2018). This type of material attracts more attention in many potential applications like heterogeneous catalysis (Wen et al. 2018), adsorption (Jiang et al. 2019), gas storage (Qasem et al. 2018), gas sensing (Wang et al. 2018a), drug delivery (Wu et al. 2017b), and photocatalysis (Xiao et al. 2019) because of their amazing characteristics, such as high porosity and large surface area, tunable functionality, and thermal and mechanical stability (Long & Yaghi 2009). Due to the low crystal density of MOFs, which have no sufficient dispersion forces to hold small molecules, an effective composite prepared of MOFs with another substrate solves this drawback (Wu et al. 2017a). Two subfamilies of MOFs are presented like zeolite imidazolate frameworks (ZIFs) and metal carboxylate frameworks (MOFs) (Orribas et al. 2016). In the metal carboxylate frameworks, the metal ions are iron (III), and terephthalic acid acts as a carboxylate linker. MIL-101(Fe) is processed in different catalytic reactions like Friedel–Crafts benzylation as it exhibits high stability and catalytic activity (Rasheed et al. 2018). One of the most effective MOFs is MIL-101 based on their unique properties such as high thermodynamically stable material with high surface area. These properties make MIL-101 good for the adsorption and separation of a wide variety of pollutants and CO_2_ gases from humid atmospheres (Mei et al. 2017). Besides, it is considered an eco-friendly material due to the central metal ion (Fe), low cost, and non-toxicity compared with other metal ions (Li et al. 2019a).

Adsorption is a physical phenomenon that occurs on surfaces and is heavily influenced by factors that include surface area, pore size, pH, solution temperature, and the kind of adsorbent and its substituent groups (Ahmad et al. 2010). Studying adsorption kinetics is crucial for determining the best adsorption system because it allows scientists to forecast the pace at which pollutants will be removed from the environment, determine how much residual adsorbate will remain in solution over time, and identify the mechanisms at play throughout the adsorption process given how straightforward and inexpensive it is compared to other methods, it is regarded as a well-developed procedure for removing dyes, pesticides, and heavy metals from wastewater (Joseph et al. 2021; Wang et al. 2020).

In this study, iron as a metal node, and 2-amino terephthalic acid was used as an organic linker in MIL-101(Fe) development. The designed adsorbent NH_2_-MIL-101(Fe) displayed a highly chelating affinity to adsorbent for pesticides and dyes attributed to the amine group that reinforced the adsorption capacity against hazardous waste from the solution. In this study, an efficient adsorbent was used to demonstrate the removal of Eosin Y dye and low and high concentrations of acetamiprid from polluted water. By using the techniques of FT-IR, XRD, XPS, SEM, EDX, TEM, and BET, samples were characterized and investigated. Other parameters were also studied, like initial concentrations of adsorbate, contact time, the initial pH of solutions, adsorbent dose, and temperature.

## Experimental

### Materials

All compounds’ substances and solvents which are applied in the adsorption studies were of the analytical grade, and none of them required further purification before usage. Ferric chloride hexahydrate (FeCl_3_.6H_2_O), 2-amino terephthalic acid (NH_2_-H_2_BDC), and Eosin yellow were purchased from Alfa Aesar. N, N-dimethyl formamide (DMF), and absolute ethanol were purchased from EDWIC, while terephthalic acid was acquired from ADVENT business. Pure acetamiprid pesticide (assay 96%) was obtained from Jiangsu Fengshan Group Co., LTD – China. Stock solutions of ATP pesticide and EY dye solutions were prepared via dist. purified water by a Milli-Q system.

### Synthesis of MIL-101(Fe) and NH_2_-MIL-101(Fe)

**Fig. 1:**
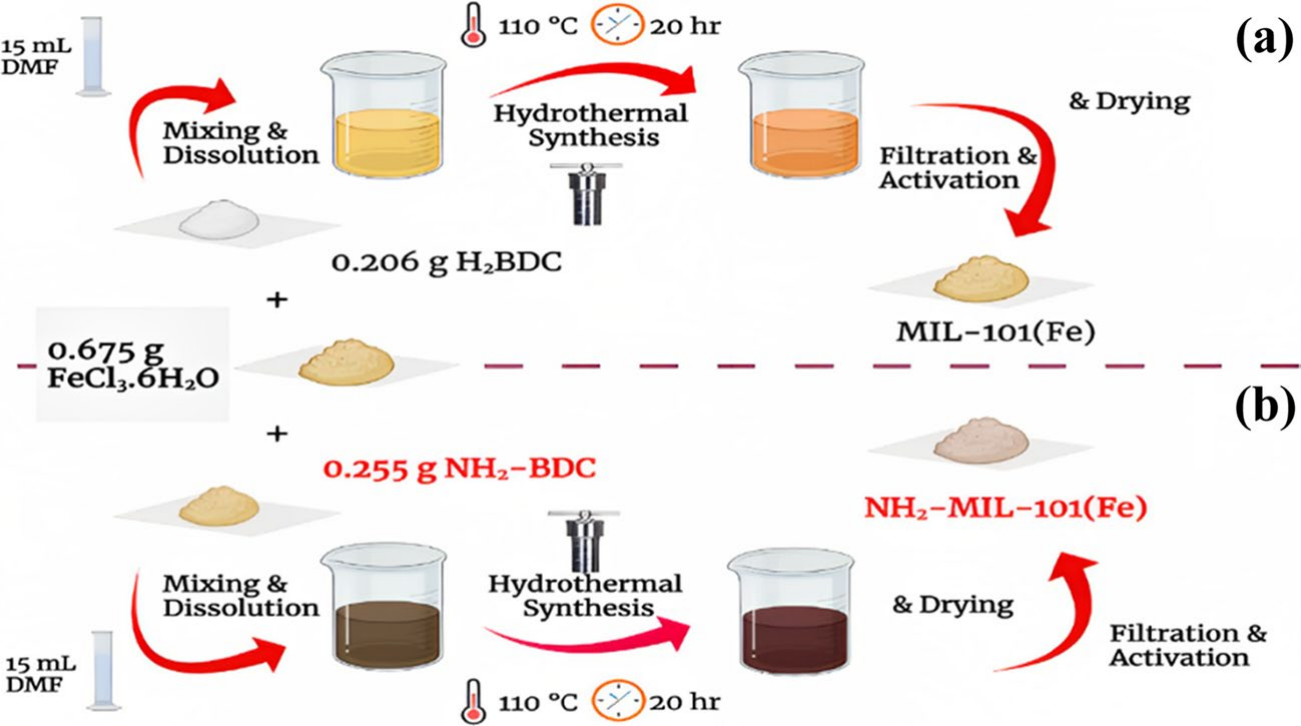
The schematic synthesis of MIL-101(Fe) and NH2-MIL-101(Fe) is shown in **a** and **b**

MIL-101(Fe) was synthesized using a solvothermal method explained elsewhere (Skobelev et al. 2013) as shown in Fig. 1. Briefly, 15 mL of DMF was used to dissolve 0.675 g (2.45 mmol) FeCl_3_6H_2_O and 0.206 g (1.24 mmol) BDC. Then the solution was moved to a Teflon autoclave made of stainless steel and sealed, for 20 h at 110 °C in a traditional oven. After centrifuging the powder, it was three times cleaned in DMF and hot ethanol before being left to dry overnight at 80 °C (Zorainy et al. 2021). The same precedence is applied in this instance synthesis of NH_2_-MIL-101 (Fe) but the linker changed to NH_2_-BDC 0.217 g)1.2 mmol).

### Batch equilibrium studies

The 50 mg of adsorbent in the 50 mL of pesticide/dye solutions was used in batch adsorption experiments. In the cases of the pesticide and dye, the solutions were churned for 120 and 140 min, respectively, after being suspended using ultrasonic technology for a few minutes. Using diluted NaOH and HCl solutions, the ideal pH of solutions was adjusted. Applying Eqs. (1) and (2), the removal % and adsorption capabilities were assessed.

1 $${\mathrm{q}}_{\mathrm{e}}=\frac{\left({\mathrm{C}}_{\mathrm{i}}-{\mathrm{C}}_{\mathrm{e}}\right){\mathrm{V}}_{\left(\mathrm{L}\right)}}{{\mathrm{m}}_{\left(\mathrm{g}\right)}}$$

2 $$\mathrm{\% Removal}=\frac{\left({\mathrm{C}}_{\mathrm{i}}-{\mathrm{C}}_{\mathrm{e}}\right)}{{\mathrm{C}}_{\mathrm{i}}}\times 100$$

where V_(L)_ represents the solution's volume, ${\mathrm{C}}_{\mathrm{i}}$ is the adsorbate concentration initially (mg. L^−1^), ${\mathrm{C}}_{\mathrm{e}}$ in balanced with the adsorbate concentration (mg.L^−1^), and ${\mathrm{m}}_{(\mathrm{g})}$ represents the dosage of adsorbent.

### Kinetic studies

To ensure that investigate of the impact of the contact time, 50 mL of ATP and EY dye with different concentrations was added to 100 mL alongside 0.05 g of an absorbent at 298 K while stirring. At time intervals, after 5, 10, 15, 30, 45, 60, 75, 90, 105, 120, 180, and 240 min, the aliquots were collected. To separate the solid from the supernatant, the aqueous solutions were diluted and centrifuged in Falcon tubes. The optimum pH of ATP and EY dye adsorption was adjusted by making adjustments with solutions of HCl and NaOH. Also, the impact of the adsorbent dosage was carried out with various masses of NH_2_-MIL-101(Fe) (0.2, 0.6, 1.0, 1.4, 1.8, and 2 g.L^−1^). The ATP pesticide and EY dye concentrations in supernatants for all experiments were measured by HPLC–DAD, and UV–visible spectrophotometer, respectively, using Eq. (1).

### Characterization

A record of the synthesized catalysts' crystal structure and phase purity was made at room temperature in the 2θ range from 4 to 70^o^ via X-ray diffraction (XRD) using a Bruker axs Co D8 Discover, beside Cu-K radiation (40 kV, 40 mA); Germany. The surface morphology and crystal size spectroscopy (FTIR) were noted on a spectrometer (Thermo Scientific Nicolet iS10 Spectrometer) applying the typical KBr disc. In order to determine compositions of elemental and chemical valance referred to the fabricated frameworks, X-ray photoelectron spectroscopy (XPS, Thermo Fisher Scientific**™** K-Alpha**™**, USA) beside monochromatic X-ray Al K-alpha radiation -10 to 1350 eV, Al-Kα micro-focused monochromator performed at an energy range up to 4 keV, during pressure 10^–9^ m bar the spot size was 400 µm along with full spectrum pass energy 200 eV and at narrow spectrum 50 eV was employed. A scanning electron microscope (SEM, JEOL JSM-6510LV, Japan) analyzed the surface morphology and crystal size of the prepared MOFs. Energy-dispersive X-ray spectroscopy (EDS) X-MaxN-20 (Oxford Instruments) was used to conduct an elemental analysis with high spatial resolution. A transmission electron microscope (TEM) (L120C—TEM—ThermoFisher—Europe) operating at 120 kV accelerating voltage was used to examine the microstructural properties of the samples. Brunauer–Emmett–Teller (BET) method was used to determine the specific surface area analysis was determined. The N_2_ physical absorption–desorption isotherms measurements were performed at 273 K applying Belsorb III equipment, Japan. The samples were evacuated at 150 °C before measurement. The remaining concentration in supernatants was evaluated using HPLC–DAD (high-pressure liquid chromatography-diode array detector Agilent Technologies 1200 series). A YP5 B C18 (125 mm × 4 mm) analytical column was utilized. 1.0 mL.min^−1^ is the rate of flow and A 30 L injection volume was used. The isocratic elution circumstances were acetonitrile/water (50:50, v/v); wavelength, 255 nm. A UV–visible spectrometer (UV-2000, UNICO, USA) was used to measure the light absorption qualities. The results for the UV–vis absorption spectra were acquired within the 200–800 nm test range.

## Results and discussion

### Design and characterization of MIL-101(Fe) and NH_2_-MIL-101(Fe)

**Fig. 2:**
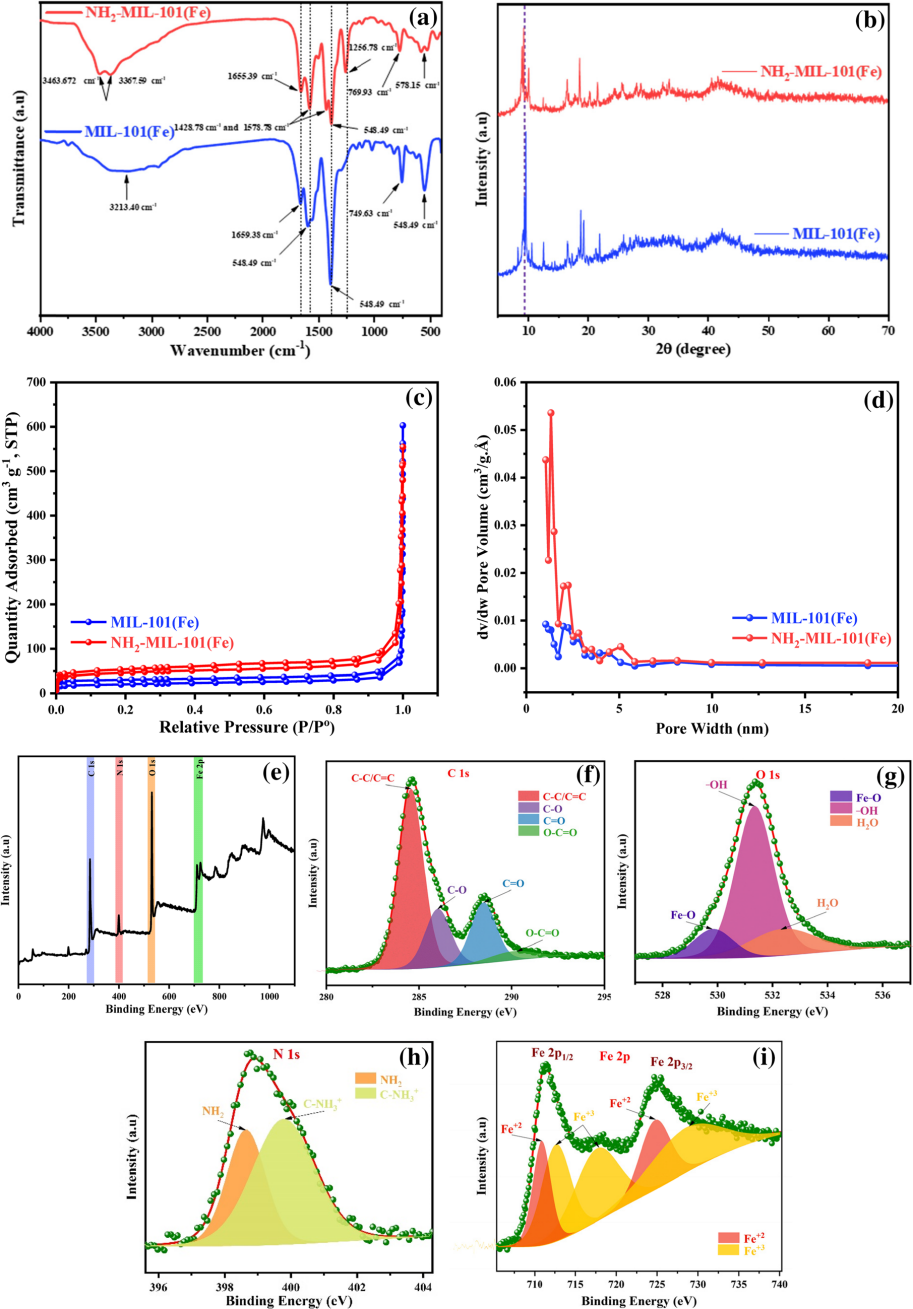
**a** FT-IR and **b** XRD of MIL-101(Fe) and NH2-MIL-101(Fe). **c** The BET-specific surface area of MIL-101(Fe) and NH2-MIL-101(Fe) isotherm. **d** Pore size distribution of MIL-101(Fe) and NH2-MIL-101(Fe) (BET). **e** Full survey XPS of NH2-MIL-101(Fe) and HR-XPS of **f** C1s, **g** O 1 s, **h** N 1 s, and **i** Fe 2p

NH_2_-MIL-101(Fe) and MIL-101(Fe) infrared spectra are displayed in Fig. 2a. As a result, the benzene-carboxylates were primarily visible in the distinctive IR spectra of MIL-101(Fe). MIL-101(Fe) spectrum displays a stretched band that is symmetrical and assigned to 3213 cm^−1^ attributed to $-$ COOH vibration (Gecgel et al. 2019a). A band at 1659.38 cm^−1^ shows the presence of DMF molecules (Wu et al. 2013). The bands at 1593 and 1388 cm^−1^ were allocated to the C $=$ O and C $-$ C vibrational modes (Xie et al. 2017). The band at 749 cm^−1^ was associated with the benzene ring in the frameworks (Wang et al. 2018b). Furthermore, the observable band at 578 cm^−1^ was allocated to the Fe–O vibration (El-Bendary et al. 2021). NH_2_-MIL-101(Fe) exhibited the same characteristic bands of MIL-101(Fe) not including the bands at 3463 and 3367 cm^−1^, which enhances the symmetric and asymmetric amine of amino terephthalic acid (Gecgel et al. 2019a). Additionally, the band assigned to 1256 cm^−1^ is attributed to the extending approach to aromatic carbon C $-$ N bonding (Xie et al. 2017).

Crystalline structures of the fabricated frameworks were distinguished by XRD as illustrated in Fig. 2b. Both fabricated MILs illustrated the main characteristic peak placed at 2θ of $\approx$ 9.2°. XRD pattern of the blank MIL-101(Fe) displayed precisely defined diffraction peaks situated in 2θ of 12.6°, 16.7°, 18.7°, and 21.9° (Zhou et al. 2020). MIL-101(Fe) and NH_2_-MIL-101(Fe) showed comparable peals in their crystal structures (Xie et al. 2017). As a consequence, both frameworks have the same crystal structure, and the successful preparation is confirmed to match the previously published diffraction pattern (Wang & Li 2015).

Validating the surface composition and chemical makeup of the as-synthesized substances was done by an investigation of the XPS analysis. A comprehensive survey scan of NH_2_-MIL-101(Fe) reveals the presence of C 1 s, N 1 s, and O 1 s with Fe 2p and Cr 2p metals, respectively, confirming the accomplishment of MOF formation which shown in Fig. 2 e, f, g, h, and i. Four peaks at ranges of 284.6, 285.9, 288.2, and 291.1 eV may be seen in the HR-XPS of C 1 s, which are attributed to C–C (sp2 and sp3), C-O, C = O, and O-C = O, correspondingly (Li et al. 2019b). At around 530.0, 531.4, and 533.0 eV, respectively, the O 1 s deconvoluted into three peaks, each of which had a MIL attributed to H_2_O, metal-O, and -OH (Zhang et al. 2021). Concurrently, the presence of an amine group in the organic linker structure resulted in the division of the N 1 s peak into two contributions denoting which represent the amino group (C $-$ NH_2_) and the protonated N species (C $-{\mathrm{NH}}_{3}^{+}$) (Solís et al. 2021). At 712.0 and 725.0 eV, respectively, the HR-XPS of the Fe 2p spectrum revealed the major peaks of Fe 2P_1/2_ and Fe 2p_3/2_. Fe^+3^ is responsible for the peaks at 714.0, 718.4, and 729.8 eV, while Fe^+2^ is responsible for the peaks at 711.1 and 724.7 (Quan et al. 2019).

**Fig. 3:**
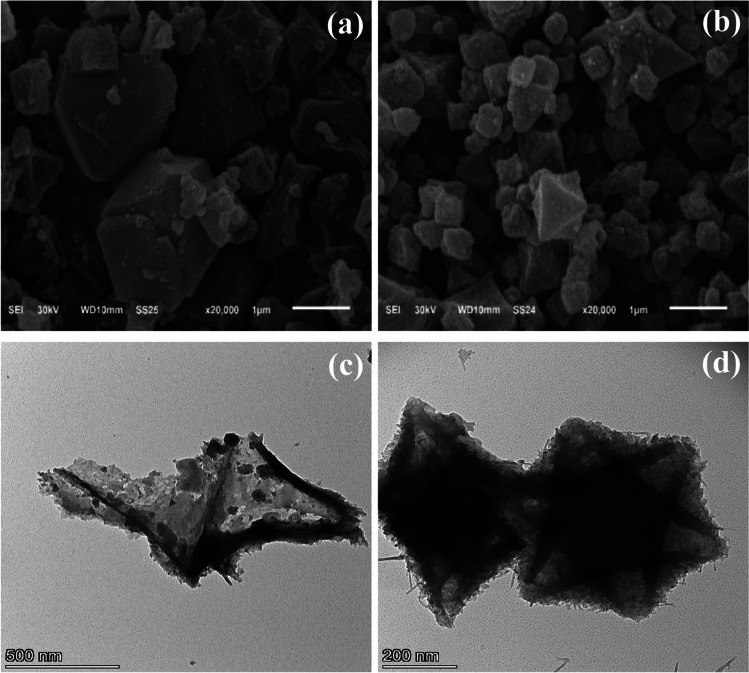
SEM images of **a** MIL-101(Fe) and **b** NH2-MIL-101(Fe) and TEM images of **c** MIL-101(Fe) and **d** NH2-MIL-101(Fe)

The SEM and TEM images of the prepared MIL-101(Fe) and NH_2_-MIL-101(Fe) are shown in Fig. 3. SEM analysis was employed to determine differences in the surface morphology of the manufactured frameworks. Figure 3 a and b shows the as-prepared framework particles with unique microstructures and morphologies of the metal-dielectric structure. It is obvious that both MIL-101(Fe) and NH_2_-MIL-101(Fe) materials have a loose and uniform polyhedron structure with an average diameter of 0.3–1 μm and 1–2 μm (Fig. S10.a and b), which matches previous reports in the literature (Boontongto & Burakham 2019, Chen et al. 2021). The crystallite size (D) was found to be 0.065648–0.000159 μm as shown in Table S1, and the particles are regular octahedrons as shown in Fig. S10. In addition, Fig. S11 illustrates the elemental analysis of the MIL-101(Fe) and NH_2_-MIL-101 (Fe) taken from EDX. According to results confirmed The presence of C, O, Fe, and N as the only elementary components of frameworks that match with data obtained from XPS (Gecgel et al. 2019b). The unique porous morphologies of the products are characterized by TEM as illustrated in Fig. 3 c and d. The as-prepared NH_2_-MIL101(Fe) is shown to have maintained the MIL-101(Fe) original morphology. The sample surface exhibits a porous frame with a hierarchical structure, which is derived from the MOFs. The morphologies of MIL-101(Fe) and NH_2_-MIL-101(Fe) show an octahedral structure, which is similar to the Zr-based materials (Xie et al. 2017).

The surface area of NH_2_-MIL-101(Fe) and MIL-101(Fe) measured by Brunauer–Emmett–Teller (BET) is determined by the surface area analyzer (Belsorb III equipment). Figure 2 c and d displays a plot of adsorbed volume vs. relative pressure. The surface area of both frameworks is acquired through nitrogen adsorption/desorption at –77 K, respectively. The BET surface area for MIL-101(Fe) is 125 m^2^.g^−1^, which is less than the amino-MIL-101(Fe) whose surface area is 953 m^2^.g^−1^ which is in line with other reports in the literature (Dong et al. 2020; Mahdipoor et al. 2021; Shan et al. 2020). As can be seen, the surface morphology of the amino-MIL-101(Fe) (Fig. S11) is much less porous compared to MIL-101(Fe), which reduces the surface area of the MOF in the absence of the amine group. In other words, functionalization of MIL-101(Fe) with the amine group increases the surface area of MOF by about seven times, which means increasing the active sites for the absorption of environmental pollutants (Rahmani et al. 2022), In addition, the results showed that the mean pore diameter and total pore volume for MIL-101(Fe) and NH_2_-MIL-101(Fe) were 3.37 nm, 0.1075 cm^3^.g^−1^, 1.88 nm nm, and 0.2456 cm^3^.g^−1^, respectively.

### Batch adsorption studies

**Fig. 4:**
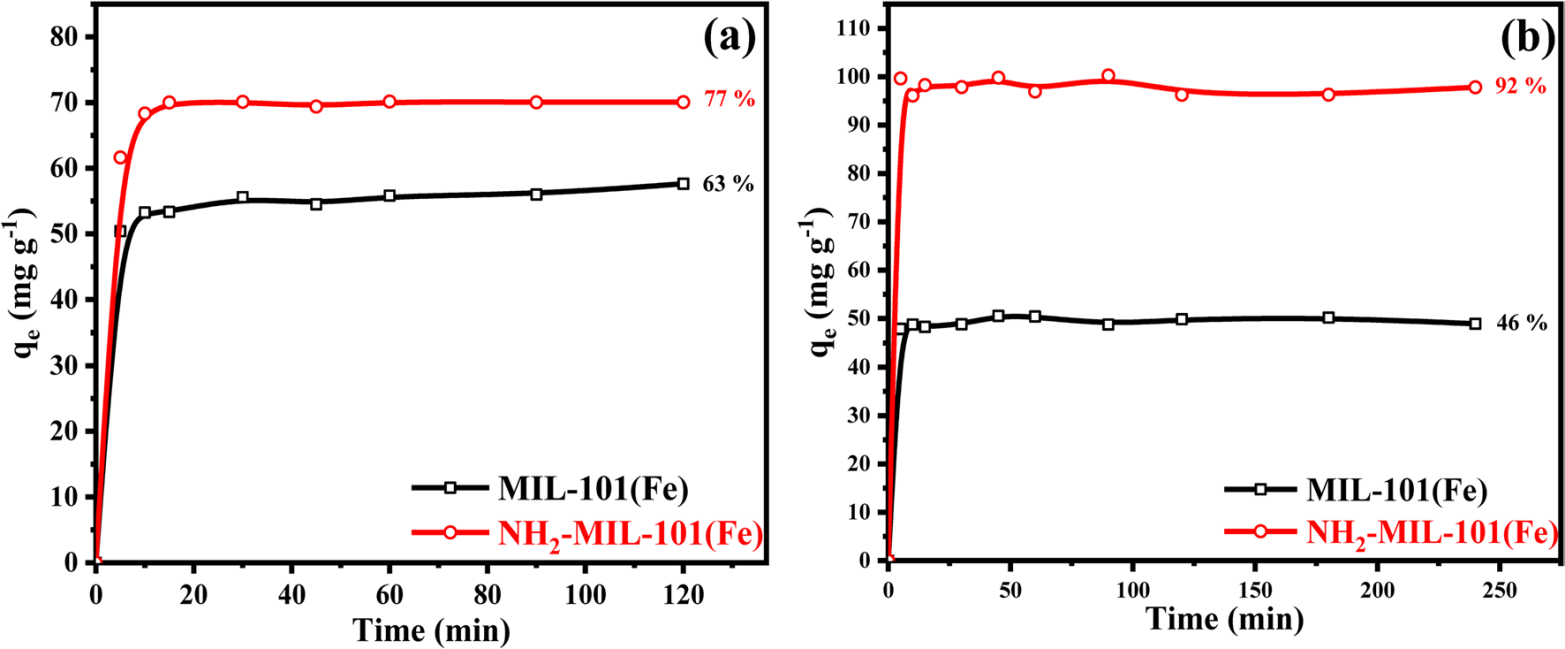
Comparison study between MIL-101(Fe) and NH2-MIL-101(Fe) for **a** ATP [conditions: initial concentration = 100 g.L-1, dose = 0.05 g, contact time = 2 h, pH 6 and temperature = 298 K]. **b** EY dye [conditions: initial concentration = 100 g.L-1, dose = 0.05 g, contact time = 4 h, pH 7 and temperature = 298 K]

The removal efficiency was studied in the presence of two Fe-based MOFs for removing ATP and EY from an aqueous solution. For ATP, both MOFs show high equilibrium capacities of 57.6 and 70.1 g.L^−1^ in 10 min of contact time for MIL-101(Fe) and NH_2_-MIL-101(Fe), respectively. in the instance of EY dye, the adsorption capacity increased from 48.9 g.L^−1^ in the instance of MIL-101(Fe) to 97.8 g.L^−1^ in 15 min contact time by utilizing amine functionalized MIL as illustrated in Fig. 4 a and b The rate of adsorption raise significantly in the instance of NH_2_-MIL-101(Fe) compared to MIL-101(Fe), it might be explained by the amino groups that it contains, which provide the adsorbate molecules more adsorption sites during the adsorption process. (Dong et al. 2020). Based on the adsorption capacity data, we can observe that the adsorption capacity of NH_2_-MIL-101(Fe) is higher than MIL-101(Fe). Furthermore, the surface area of NH_2_-MIL-101(Fe) is 953 m^2^g ^−1^ which is greater than that MIL-101(Fe) (Dong et al. 2020; Rahmani et al. 2022; Shan et al. 2020; Xu et al. 2020).

**Fig. 5:**
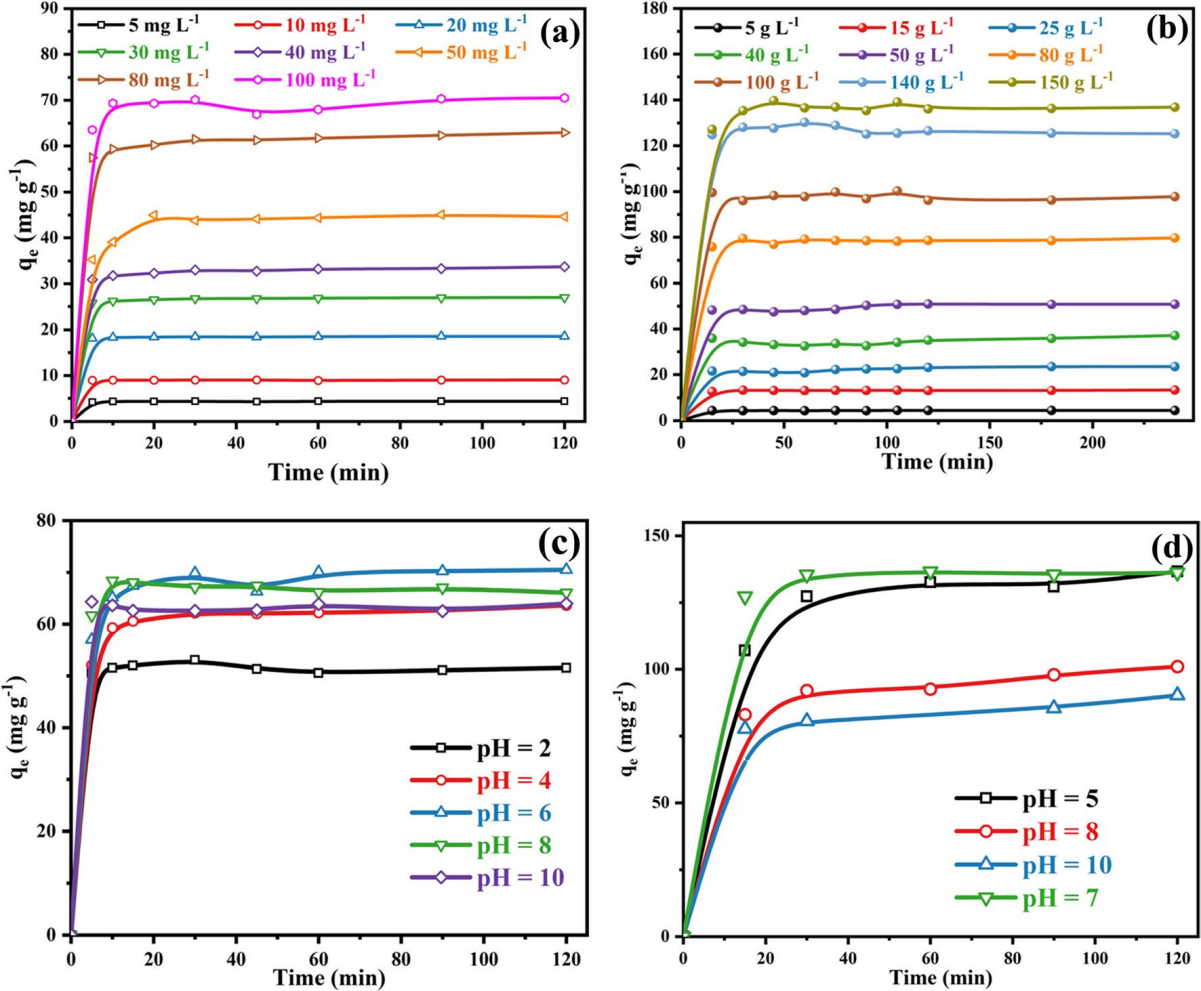
Effect of initial concentration for adsorption of **a** ATP [conditions: initial concentration = 5–100 g.L-1, dose = 0.05 g, contact time = 2 h, pH 6 and temperature = 298 K]. **b** EY dye [conditions: initial concentration = 5–150 g.L-1, dose = 0.05 g, contact time = 4 h, pH 7 and temperature = 298 K]. Effect of solution’s pH on adsorption of **c** ATP [conditions: initial concentration = 100 g.L-1, dose = 0.05 g, contact time = 2 h and temperature = 298 K]. **d** EY dye [conditions: initial concentration = 150 g.L-1, dose = 0.05 g, contact time = 2 h and temperature = 298 K]

For the adsorption experiments, different initial concentrations were performed between 5 and 100 mg.L^−1^ under the same conditions of temperature, dose, and pH. Increased dye and pesticide initial concentration leads to an increase in equilibrium adsorption capability. Nevertheless, for NH_2_-MIL-101(Fe), it was discovered that at a low concentration (between 5 and 30 mg. = L^−1^), as the initial concentration of ATP rises, the adsorption capacity reached equilibrium rapidly, and the speed slowed when the concentration level was raised Fig. 5a. This is due to the fact that a high adsorbate concentration can supply the required driving force to get over the adsorbate mass transfer barrier between the aqueous and solid phases. However, EY’s adsorption was investigated at various initial concentrations of 5–150 g.L^−1^ under room temperature. Figure 5b illustrated that NH_2_-MIL-101(Fe) exhibited excellent removal of EY dye with an equilibrium concentration of 136.8 g.L^−1^. More than 90% of EY dye was eliminated from the aqueous solution for the first 15 min in the case of all initial concentrations. After that, it faded off with progressively more time of contact. This behavior is because all of the active sites on the adsorbent were unoccupied at first and was high concentration of dye. Because of the possession of the active sites by dye molecules, the number of active sites was reduced, and the rate of sorption decreased as time passed (Nazir et al. 2022). After the equilibrium was attained, the adsorption process was not feasible due to the possible monolayer of dye formation onto the adsorbent surface.

The concomitant effect of solution pH within the scope of 2–10 on the removal capacity of ATP was evaluated and the obtained contour graphs are displayed in Fig. 5c. The efficiency of removal of pesticides reached the maximum value at a neutral medium and reduced at lower and higher pH values. Because ATP has a low pK_a_ of 0.7, it is found in aqueous solutions in its anionic form and its charge is unaffected by changes in solution pH (Dolatabadi et al. 2021). Therefore, at a lower pH, there is a positive charge on the adsorbent surface, and at a higher pH, negatively charged (Dolatabadi et al. 2021). The removal efficacy reduces at higher pH levels due to the electrostatic repulsion force among adsorbent and ATP molecules. At very low pH (pH = 2), the removal efficiency reduces, which is due to the as-synthesized frameworks that may undergo hydrolysis in a highly acidic medium. Besides, although NH_2_-MIL-101(Fe) was stable in both acids and water, it degraded quickly, especially under strong basic conditions. The weak acidic to neutral conditions of pH values should thus be maintained (pH ~ 6) to achieve a high adsorption capacity of ATP (Li et al. 2020b; Ma et al. 2022).

For EY dye removal, the pH range was between 5 and 10 and the optimum pH was between 5 and 7 as depicted in Fig. 5d. This resulted from the protonated amine group of NH_2_-MIL-101(Fe) interacting beside the dye anionic group during the adsorption process, which led to the creation of more cationic amines (Du et al. 2008). Furthermore, due to the higher electrostatic interactions, cause the dye molecules $(\mathrm{D}-\mathrm{COONa})$ to be absorbed by the frameworks $(\mathrm{R}-{\mathrm{NH}}_{2})$ to a greater extent as shown in Eq. (3–5). Therefore, the protonation of the amine group in the framework structure decreased at higher pH, and the removal capacity of EY dye decreased. The lower pH of the aqueous solution was not favored because most of the dye molecules were noticed to be precipitated and the color was changed during the adsorption of the dye.

3 $$\mathrm{R}-{\mathrm{NH}}_{2}+{\mathrm{H}}_{3}{\mathrm{O}}^{+}\to {\mathrm{R}-\mathrm{NH}}_{3}^{+}+{\mathrm{H}}_{2}\mathrm{O}$$

4 $$\mathrm{D}-\mathrm{COONa}\stackrel{{\mathrm{H}}_{2}\mathrm{O}}{\to }\mathrm{D}-{\mathrm{COO}}^{-}+{\mathrm{Na}}^{+}$$

5 $${\mathrm{R}-\mathrm{NH}}_{3}^{+}+\mathrm{ D}-{\mathrm{COO}}^{-}\to {\mathrm{R}-\mathrm{NH}}_{3}^{+}\mathrm{D}-{\mathrm{COO}}^{-}$$

**Fig. 6:**
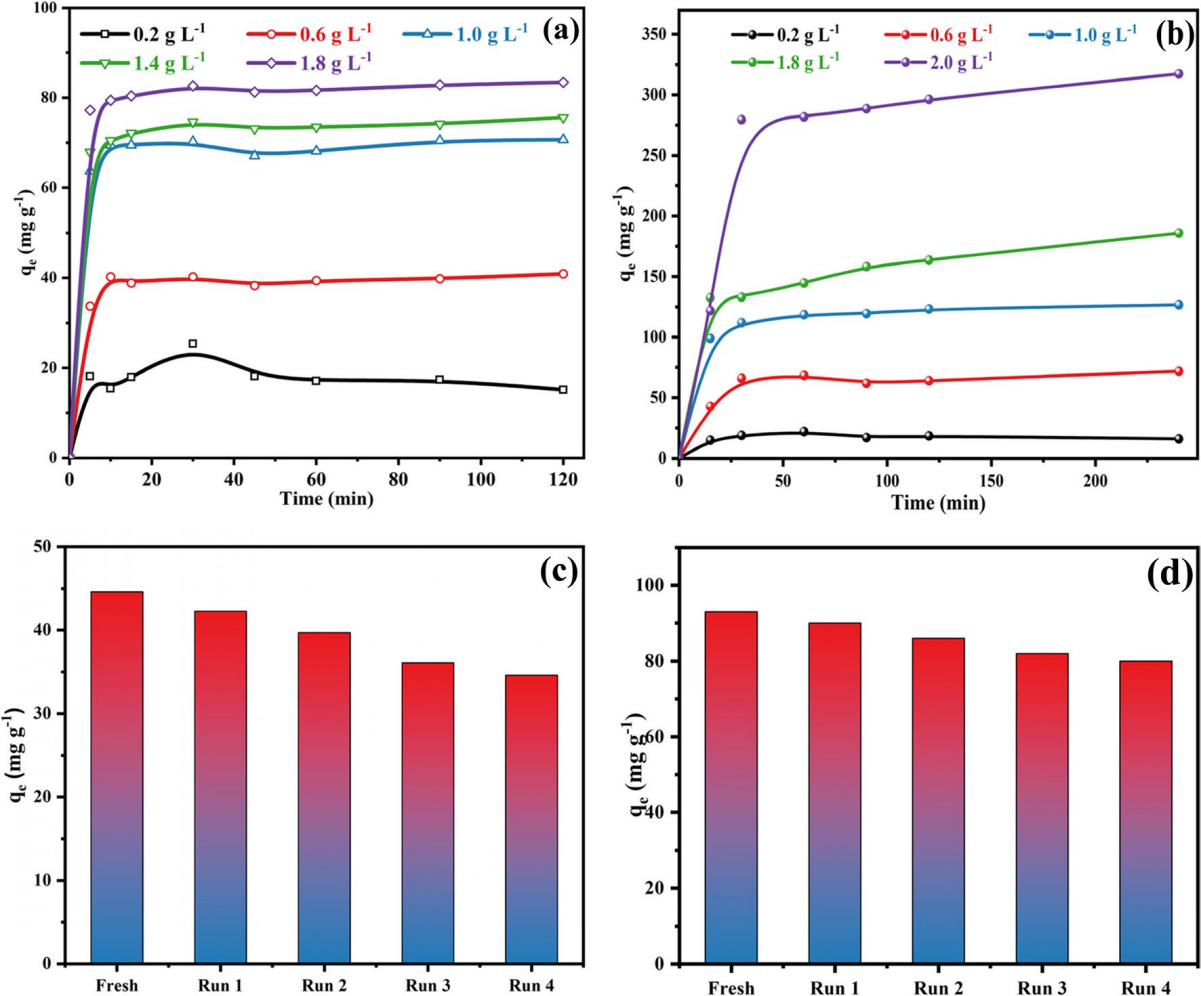
Effect of adsorbent dose for adsorption of **a** ATP [conditions: initial concentration = 100 g.L-1, pH 6, contact time = 2 h and temperature = 298 K]. **b** EY dye [Conditions: initial concentration = 500 g L-1, pH 7, contact time = 2 h and temperature = 298 K]. Effect of reusability for adsorption of **c** ATP [conditions: initial concentration = 50 g.L-1, pH 6, contact time = 2 h and temperature = 298 K]. **d** EY dye [conditions: initial concentration = 50 g.L-1, pH 7, contact time = 4 h and temperature = 298 K]

The influence of adsorbent dosage on the removal of ATP utilizing the Fe-based frameworks was examined in the presence of different masses as shown in Fig. 6a. The rate of adsorption ATP enhanced through increasing the mass of adsorbent (0.2–1.8 g.L^−1^). Additionally, the same behavior was illustrated for the adsorption of EY dye on raising the dosage (0.2–2.0 g.L^−1^). The total number of adsorption sites rises dramatically at the high adsorbent dose, which leads to a larger removal of ATP and EY dye from the aqueous solution because the adsorbent active sites are more easily accessible and can interact with the analyte (Ghiasi et al. 2022; Mohammad et al. 2020). For ATP, the removal percentage reached 80.2% in the case of 1.0 g.L^−1^ compared to 94.3% when the dosage nearly doubled to 1.8 g.L^−1^. Therefore, the removal efficiency reached the highest of 316.9 mg.g^−1^ utilizing 2.0 g.L^−1^ in the case of EY dye Fig. 6b. Therefore, considering the economic cost, the optimal adsorbent dose is 1.0 g.L^−1^ that could be applied in the ensuing study.

**Fig. 7:**
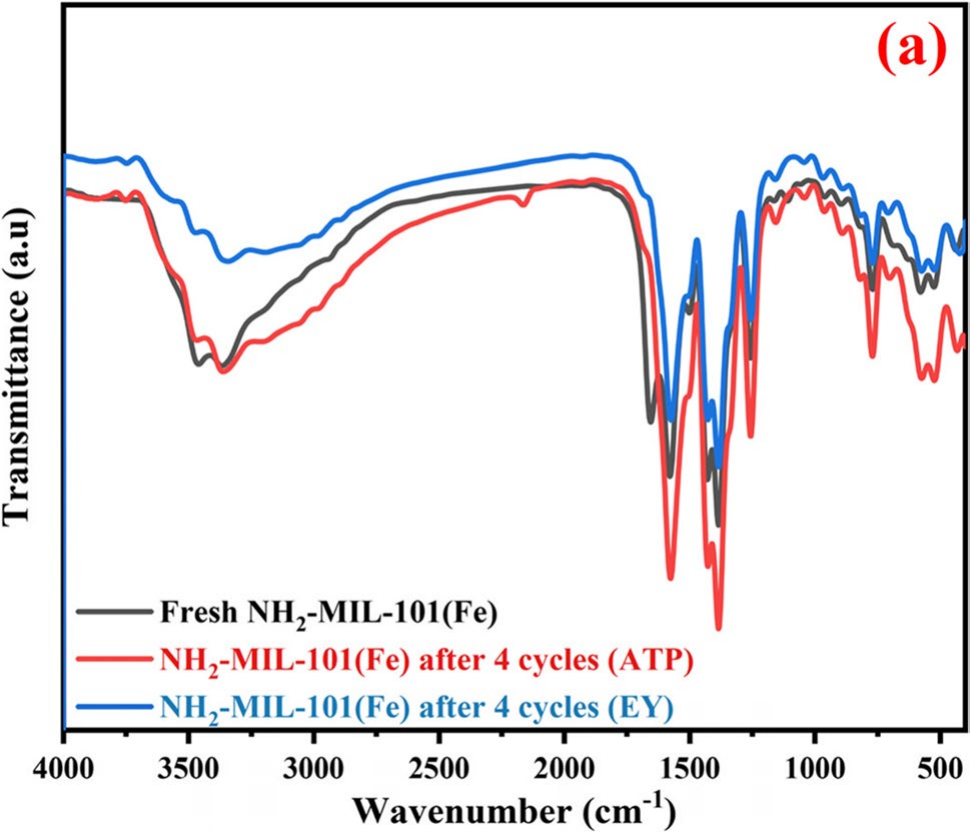
**a** FT-IR spectra of NH2-MIL-101(Fe) before and after adsorption of (a) ATP and (b) EY

The capacity to regenerate and reuse used adsorbents is crucial for cost-effective industrial applications. The reusability studies were carried out for ATP and EY dye for four cycles to confirm the industrial use of NH_2_-MIL-101(Fe). Upon completion of each cycle, the used adsorbent was extracted from the solution using a centrifuge, and it was thereafter repeatedly cleaned using distilled water and heated ethanol. Next, the repurposed adsorbent was dried at 80 °C and reused for the next cycles. For the initial cycle, the adsorption capacities were 96.5% and 93.4% for ATP and EY dye, as shown in Fig. 6c and d. After the fourth cycle, slight decreases in the removal percentages were noticed after four cycles. Moreover, Fig. 7a demonstrates that the FT-IR spectrum of the materials has no significant change. This means that there is no significant difference between the chemical bond and crystal structure of the sample before and after the reaction. Therefore, the NH_2_-Fe-MILs material has a strong reusability performance. Table S2 clearly shows that NH_2_-Fe-MILs material is an effective heterogeneous catalyst for the removal of ATP and EY dye from industrial wastewater which means it has the potential ability to remove other organic pollutants in industrial wastewater, and the XRD characterization of the material after four cycles showed that the peak intensity of the material was decreased. However, their crystalline structure was found to remain moderately intact after the repeated cycles (Fig. S13.). The use of the TEM technique revealed that after four cycles consecutive, there were no changes observed in the structure or crystallinity of the nanocomposite following the adsorption process. This observation suggests that the composite maintained high stability, as indicated in Fig. S12.

Figure 7a displayed the FTIR spectrum of the recycled frameworks after four cycles in the case of ATP and EY dye. The spectrum illustrates no considerable changes in the functional groups or their positions. This confirms that the prepared NH_2_-MIL-101(Fe) is an effective adsorbent in the removal of organic pollutants from aqueous solutions after various cycles.

The XRD patterns for MIL-101(Fe) and NH_2_-MIL-101(Fe) feature very similar peaks, as depicted in Fig. S13. This is because terephthalic acid, which is used to synthesis MIL-101(Fe), and 2-amino terephthalic acid, which is used to synthesis NH_2_-MIL-101(Fe), have similar structures. As a result, the products maintain similar crystal structures (Liu et al. 2022). Furthermore, the XRD pattern showed no discernible shift before or after adsorption, suggesting that the adsorbents’ structures were stable.

Utilizing transmission electron microscopy (TEM), an advanced imaging technique capable of high-resolution analysis provided crucial insights into the structural integrity of the nanocomposite. Through TEM analysis, it was established that there were no discernible alterations observed in the structural composition or crystalline characteristics of the nanocomposite subsequent to the adsorption process. This observation underscores the remarkable stability exhibited by the composite material. The findings, depicted graphically in Fig. S12, serve to highlight the robustness of the nanocomposite following its interaction with the adsorbate.

As listed in Table S3 and Table S4, a comparison of the ATP insecticide and EY dye adsorption in previous studies using different adsorbents with our work is illustrated. Remarkably, a high adsorption capacity was attained at a moderately brief time using MIL-101(Fe) and NH_2_-MIL-101(Fe) throughout this study.

### Adsorption isotherm

In order to assess an adsorbent's sorption capacity and comprehend how the adsorbent binds and interacts with the adsorbate, it is crucial to use the adsorption isotherm method. This article aims to demonstrate the equilibrium nature of adsorption by examining many adsorption isotherm models. The constant parameters for each model were determined by fitting the experimental data with the Freundlich, Langmuir, D–R, Redlich-Peterson, and Temkin isotherm equations.

#### Freundlich and langmuir model

Adsorbent utilization can be improved by using the isotherm of adsorption; this is a general equilibrium relation that describes how an adsorbate and the binding sites scattered over a solid surface interact (Adly et al. 2021). Adsorption isotherm equations for the experimental findings were obtained by applying Langmuir and Freundlich (Xie et al. 2017).

Because of the adsorbent heterogeneous surface that exhibits multilayer adsorption, the Freundlich pattern is appropriate for application. The maximal adsorption capacity in this model is not known with certainty, since there are different adsorption sites with varying energies. The model of Freundlich is expressed as Eq. 6:

6 $${\mathrm{logq}}_{\mathrm{e}}=\mathrm{ log}{\mathrm{K}}_{\mathrm{f}}+\frac{1}{\mathrm{n}}{\mathrm{logC}}_{\mathrm{e}}$$

where, K_f_ ($\mathrm{L}$.mg^−1^) and the constants of Freundlich that correspond to the adsorbent capacity for adsorption are represented via $\mathrm{n}$, and adsorption intensity is represented as 1/n. The Freundlich coefficient, also known as the heterogeneity coefficient, is 1/n and it describes the deflection from linear adsorption. The sort of isotherm is either favorable (1/n < 1) or unfavorable (1/n > 2) based on the value of 1/n.

Because the surface is energetically homogenous, all adsorption sites are assumed to be equally active, and a monolayer surface coverage forms without any interaction among the adsorbed molecules, the Langmuir model is developed (Araújo et al. 2018). Equation 7 represents the Langmuir model:

7 $$\frac{{\mathrm{C}}_{e}}{{\mathrm{q}}_{\mathrm{e}}}= \frac{1}{{{\mathrm{K}}_{\mathrm{L}}\mathrm{q}}_{\mathrm{m}}} + \frac{1}{{\mathrm{q}}_{\mathrm{m}}}{\mathrm{C}}_{\mathrm{e}}$$

where ${\mathrm{C}}_{\mathrm{e}}$ is the concentration of solute at equilibrium (mg.L^−1^), ${\mathrm{q}}_{\mathrm{e}}$ is solute adsorbed quantity per mass of adsorbent (mg.g^−1^), ${\mathrm{K}}_{\mathrm{L}}$ (L.mg^−1^) is the adsorption bonding energy-related Langmuir adsorption constant, and ${\mathrm{q}}_{\mathrm{m}}$ is the capability of Single-layer Langmuir adsorption (mg.g^−1^). Furthermore, this model posits a one-to-one relationship, wherein a single adsorbate can be drawn to each adsorptive site.

**Fig. 8:**
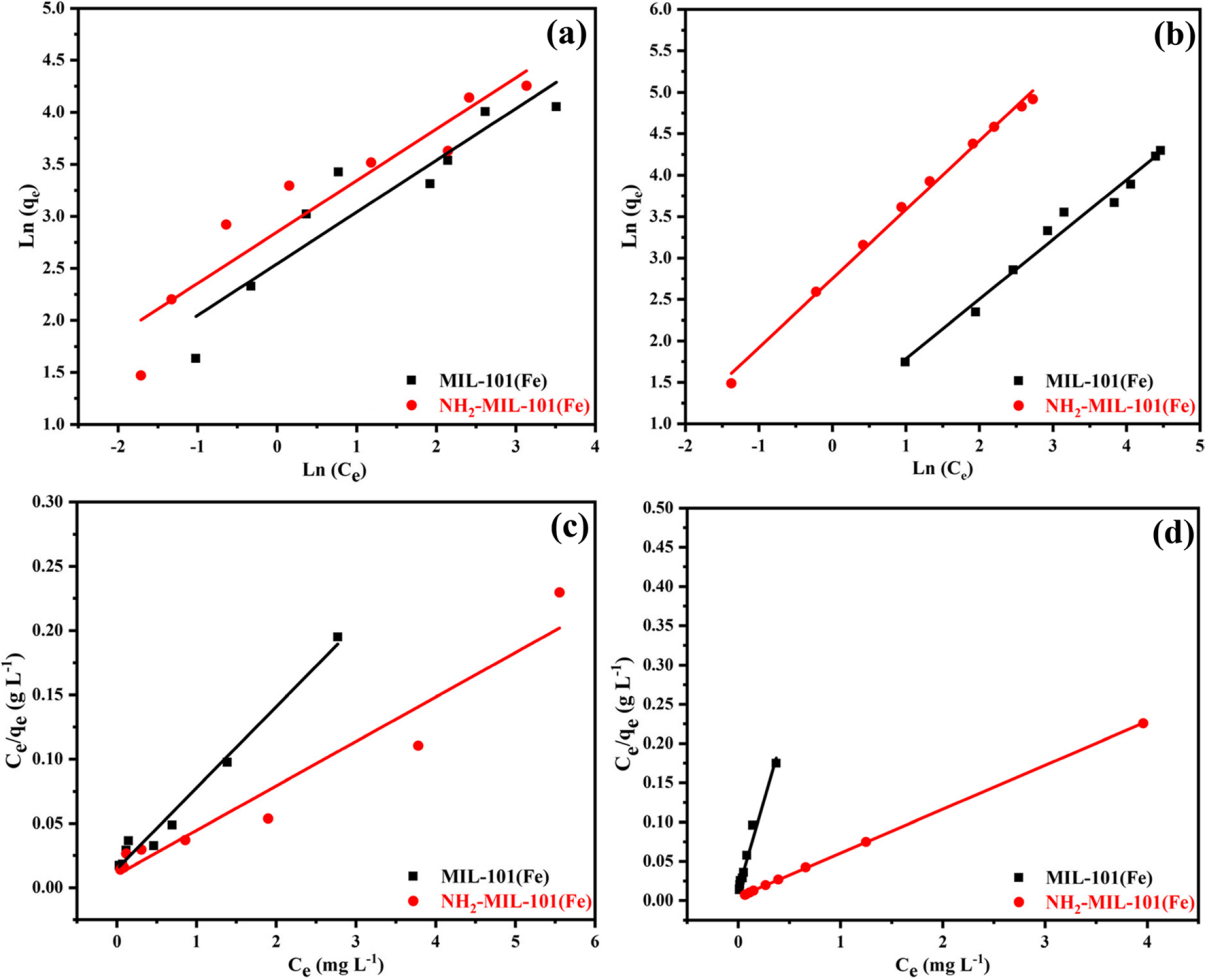
Freundlich and Langmuir adsorption isotherms for the adsorption of ATP (**a**, **c**) and EY (**b**, **d**) on NH2-MIL-101 adsorbent

Table 1 provides the linear fitting process findings for Freundlich and Langmuir constants. The correlation coefficients found using an equation for Langmuir were greater than those using the equation for Freundlich. Therefore, as shown in Fig. 8, the model of Langmuir model performed better than the model Freundlich, showing that the adsorption of ATP insecticide and EY dye on the surface of NH_2_-MIL-101(Fe) was restricted to the creation of monolayers and that there were no interactions between them; the interaction between adsorbent and adsorbate was physical adsorption processes (Liu et al. 2021).

**Table 1:** Adsorption isotherms constants for the adsorption of ATP and EY on NH_2_-MIL-101 adsorbent

Model	Pollutant	ATP		EY dye	
	Adsorbent	MIL-101(Fe)	NH_2_-MIL-101(Fe)	MIL-101(Fe)	NH_2_-MIL-101 (Fe)
Freundlich	${\mathrm{K}}_{\mathrm{f }}(\mathrm{L}.{\mathrm{mg}}^{-1})$	12.73813	17.28951	2.90432	15.677
	1/$\mathrm{n}$	0.4968	0.4944	0.7192	0.8318
	${\mathrm{R}}^{2}$	0.8701	0.8916	0.9795	0.9956
Langmuir	${\mathrm{K}}_{\mathrm{L}}(\mathrm{L}.{\mathrm{mg}}^{-1})$	0.231378	0.28902	0.03053	0.07857
	${\mathrm{q}}_{\mathrm{m}}(\mathrm{mg}.{\mathrm{g}}^{-1})$	68.49315068	100	72.4638	227.273
	${\mathrm{R}}^{2}$	0.9812	0.93622	0.9795	0.9999
	${\mathrm{Q}}_{\mathrm{max}}(\mathrm{mg}.{\mathrm{g}}^{-1})$	37.60	12.924	37.50	13.00
D R	${\mathrm{K}}_{\mathrm{d}}({\mathrm{mol}}^{2}.{\mathrm{kJ}}^{-2})$	2.026E-7	3.31801E-8	3.40882E-6	5.73259E-8
	$\mathrm{E}(\mathrm{KJ}.{\mathrm{mol}}^{-1})$	1.570	3.881	0.382	2.953
	${\mathrm{R}}^{2}$	0.86235	0.63401	0.62591	0.79889
Redlich-Peterson	$\mathrm{A}(\mathrm{L}.{\mathrm{g}}^{-1})$	12.7377	12.30	2.9042	15.6766
	$\upbeta$	0.50316	0.50604	0.28075	0.16818
	${\mathrm{R}}^{2}$	0.87289	0.89618	0.87908	0.90269
	${\mathrm{A}}_{\mathrm{t}} (\mathrm{L}.{\mathrm{g}}^{-1})$	1.0759	1.1323	1.2000	1.3878
Temkin	${\mathrm{b}}_{\mathrm{t}} (\mathrm{J }.{\mathrm{mol}}^{-1})$	215.1417	198.3263	129.6160	74.2348
	$\mathrm{B }(\mathrm{J }.{\mathrm{mol}}^{-1})$	11.5160	12.4924	19.1147	33.3748
	${\mathrm{R}}^{2}$	0.89136	0.91393	0.88805	0.87822

Figure 8 displays the adsorption isotherm of ATP pesticide and EY dye adsorbed by MIL-101(Fe) and NH_2_-MIL-101(Fe). Table 1 displays the isotherm parameters together with the matching correlation coefficients (R^2^) of isotherm models. The adsorption capability of MIL-101(Fe) and NH_2_-MIL-101(Fe) adsorbents to ATP insecticide and EY dye was very high. As seen in Fig. 8 and Table 1, the adsorption of ATP pesticide and EY dye is described very well by both Langmuir and Freundlich isotherms. On MIL-101(Fe) and NH_2_-MIL-101(Fe), the Langmuir maximum adsorption capacities of ATP insecticide and EY dye on MIL-101(Fe) were 57.6 and 48.9 mg/g while on NH_2_-MIL-101(Fe) was 70.5 and 97.8 mg/g, respectively. The isotherm of ATP pesticide and EY dye matched better with the Langmuir model (R^2^ = 0.9812, 0.93622, 0.9795 and 0.9999) than the Freundlich model (R^2^ = 0.8701,0.8916, 0.9795 and 0.9956) for both MIL-101(Fe) and NH_2_-MIL-101(Fe). This suggests that the ATP pesticide and EY dye adsorption on MIL-101(Fe) and NH_2_-MIL-101(Fe) may be controlled by the physical adsorption. Additionally, the favorable adsorption of ATP insecticide and EY dye onto MIL-101(Fe) and NH_2_-MIL-101(Fe) was verified by the values of 1/n (0.4968, 0.4944, 0.7192, and 0.8318).

#### Dubinin-radushkevich model

**Fig. 9:**
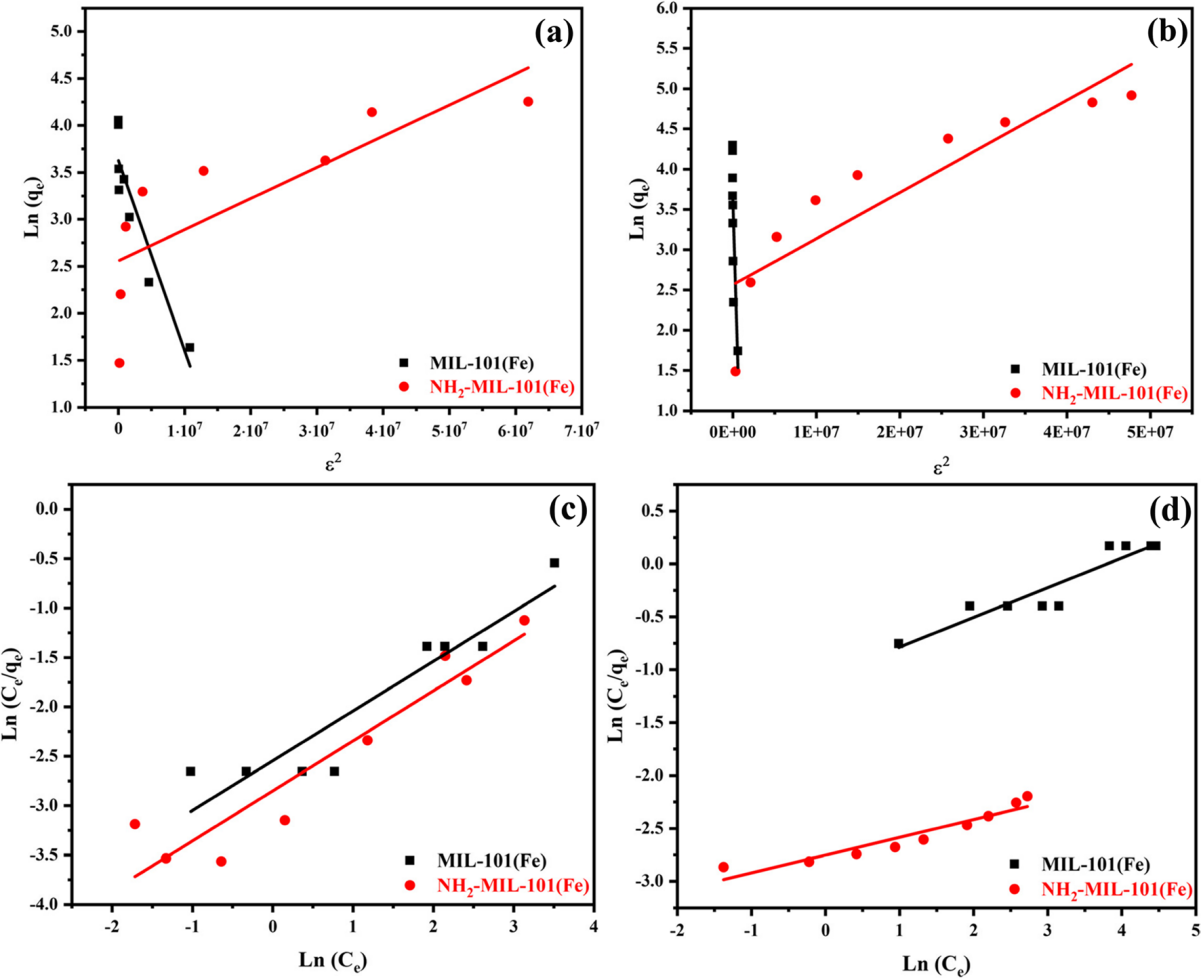
DR and Redlich-Peterson isotherms for the adsorption of MIL-101(Fe) and NH2-MIL-101(Fe) of **a** ATP and **b** EY (100 ppm)

(D-R model) was put up as an empirical isotherm to demonstrate the vapor's adsorption onto solid materials (Dubinin 1960). The D-R model is developed on the basis of Polanyi theory and the premise that the distribution of pores in an adsorbent follows the Gaussian energy distribution. It is based on the theory that the process of adsorption is pore volume filling as opposed to a layer-by-layer build-up of a film on the pore walls (Wang & Guo 2020a) (Fig. 9a and b). The D-R model that is linear can represented according to the following equation:

8 $${\mathrm{q}}_{\mathrm{e}}={\mathrm{Q}}_{\mathrm{max}}-\mathrm{exp}\left({-\mathrm{K}}_{\mathrm{D}}{\upvarepsilon }^{2}\right)$$

9 $$\upvarepsilon =\mathrm{RTln}\left(1+\frac{1}{{\mathrm{C}}_{\mathrm{e}}}\right)$$

where ${\mathrm{Q}}_{\mathrm{max}}$ (mg.g^−1^) represents the maximum amount of adsorbed substances, the constant that is associated with the computed average sorption energy E is K_D_ (mol^2^.kJ^−2^), ε (kJ.mol^−1^) is Polanyi’s potential theory-based adsorption potential calculated by Eq. (10), R is gas’ constant, and the absolute temperature is T (Mozaffari Majd et al. 2022).

Using Eq. 11 will calculate the mean free energy (E, kJ.mol^−1^).

10 $$\mathrm{E}=\frac{1}{\sqrt{2{\mathrm{K}}_{\mathrm{D}}}}$$

The index of energy consumed is denoted by the symbol E, which indicates the free energy per mole of metal ions going from infinity in solution to the sorbent surface. E is frequently used to assess whether a physical process (where E is less than 8 kJ.mol^−1^) or chemical process (8 < E < 16 kJ.mol^−1^) is dominant in the adsorption (Chabani et al. 2006).

The linear plot of ε^2^ vs ln (q_e_) is displayed in Fig. 9a and b. Table 1 displays the computed D-R constants and mean free energy for adsorption. The range of 1.570, 3.881, 0.382, and 2.953 kJ.mol^–1^ for the mean adsorption energy suggested that the adsorption process is physical. Depending on the correlation coefficient values as indicated in Table 1, the D-R isotherm model provides a good match to the experimental data.

#### Redlich-peterson isotherm model

Since it is flexible enough to be applied in both homogeneous and heterogeneous systems, the Redlich-Peterson isotherm model may be used to represent the adsorption equilibrium across a wide range of concentrations (Ayawei et al. 2017). According to (Foo & Hameed 2010, Redlich & Peterson 1959), this isotherm may be represented by the nonlinear equation Eq. (12) and the linear equation Eq. (13) and Fig. 9c and d shows this in addition to Table 1.

11 $${\mathrm{q}}_{\mathrm{e}} =\frac{{\mathrm{BC}}_{\mathrm{e}}}{1+\mathrm{A}{({\mathrm{C}}_{\mathrm{e}})}^{\upbeta }}$$

12 $$\mathrm{In}\left(\frac{{\mathrm C}_{\mathrm e}}{{\mathrm q}_{\mathrm e}}\right)=\mathrm\beta\;\mathrm{In}\left({\mathrm C}_{\mathrm e}\right)-\mathrm{In}\left(\mathrm A\right)$$

where C_e_ denotes the equilibrium concentration, $\upbeta$ denotes the Redlich–Peterson isotherm exponent, B denotes the Redlich–Peterson isotherm constant (L.g^–1^), A denotes the Redlich–Peterson isotherm constant (L.g^–1^), and q_e_ is the quantity of adsorbate in the adsorbent at equilibrium (mg. g^–1^).

Redlich-Peterson constants may be calculated by plotting ln (C_e_) against ln (C_e_/ q_e_), where β represents the slope and A the intercept (Brouers & Al-Musawi 2015).

This isotherm model is versatile enough to be used in both homogeneous and heterogeneous systems, with a linear dependence on concentration in the numerator and an exponential function in the denomination. Together, these features represent adsorption equilibrium over a wide range of adsorbate concentrations (Gimbert et al. 2008).

The R–P have also been applied to evaluate the fitting for the adsorption of ATP insecticide and EY dye (Fig. 9c and d). The calculated isotherm parameters and their corresponding coefficient of determination, R^2^, values are shown in Table 1.

#### Temkin isotherm model

Additionally, based on this model of isotherm, the impact of an indirect adsorbent and interactions of adsorbate on adsorption will cause the adsorption heat of all layers to drop linearly beside coverage. the model of Temkin isotherm is utilized to calculate the sorbent sorption capability for organic compounds (ATP pesticide and EY dye) and assumes that the adsorption heat drop is linear as opposed to logarithmic, as indicated by the Freundlich equation (Aharoni & Ungarish 1977, Ashouri et al. 2021). Typically, this paradigm is applied according to Eqs. (14) and (15) (Ashouri et al. 2021; Benjelloun et al. 2021):

13 $${\mathrm{q}}_{\mathrm{e}} =\frac{\mathrm{RT}}{{\mathrm{b}}_{\mathrm{t}}}\mathrm{ ln }({\mathrm{A}}_{\mathrm{t}})+\frac{\mathrm{RT}}{{\mathrm{b}}_{\mathrm{t}}}\mathrm{ ln }({\mathrm{C}}_{\mathrm{e}} )$$

where,

14 $$\mathrm{B}=\frac{\mathrm{KT}}{{\mathrm{b}}_{\mathrm{t}}}$$

K_R_,a_R_ g (0 < g < 1) are constants of isotherms, ${\mathrm{A}}_{\mathrm{t}}$ Temkin isotherm is the constant of binding for equilibrium that reflects the highest possible binding energy value (L.g^−1^), the positive value of constant B is 11.5160 and 12.4924 J.mol^−1^ MIL-101(Fe) (ATP and EY) 19.1147 and 33.3748 J.mol^−1^ NH_2_-MIL-101(Fe) (ATP and EY) reflects a process of endothermic calculated by Eq. (15). ${\mathrm{b}}_{\mathrm{t}}$ is the constant of Temkin isotherm that represents the heat of adsorption (J.mol^−1^), C_e_: equilibrium concentration (mg.L^−1^),${\mathrm{q}}_{\mathrm{e}}$: the adsorbate content of the adsorbent at equilibrium (mg.g^−1^); R is the constant of universal gas (8.314 J mol^−1.^K^−1^) and the absolute temperature is T (K).

**Fig. 10:**
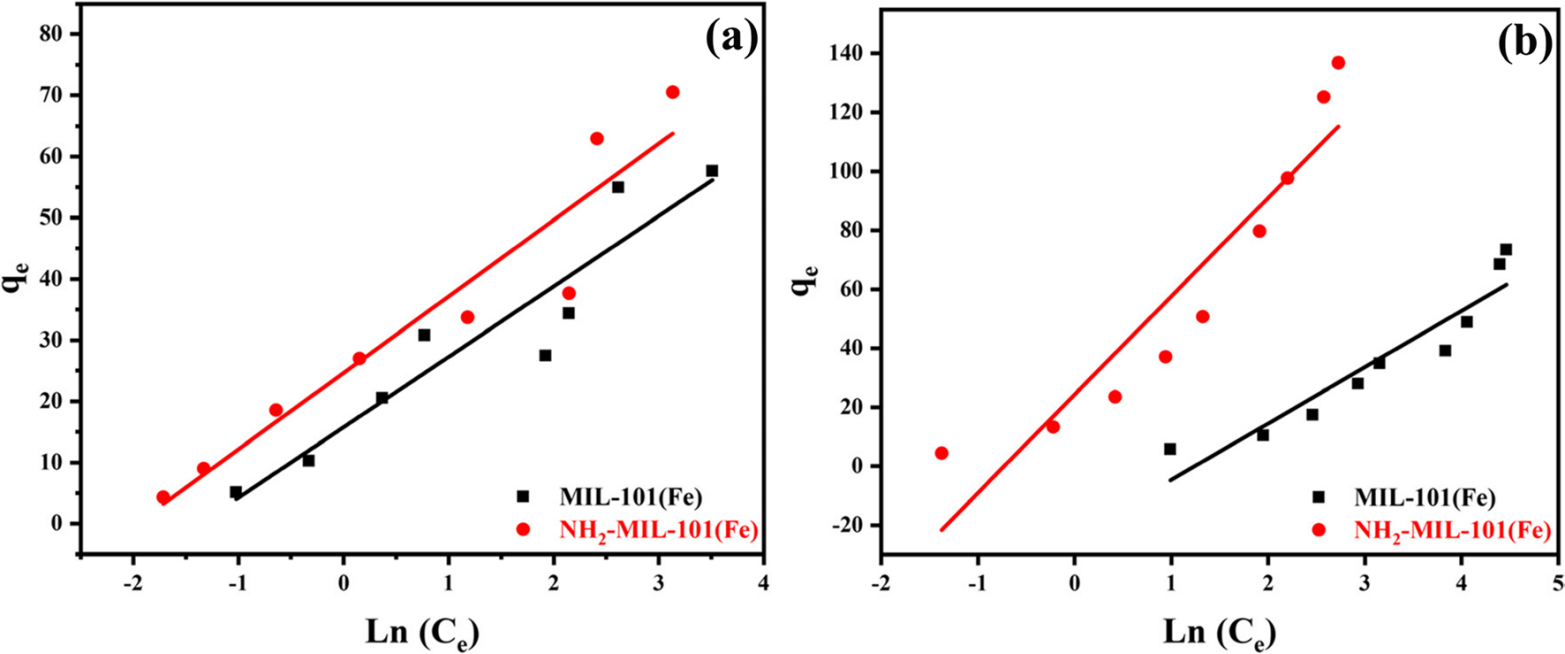
Temkin of MIL-101(Fe) and NH2-MIL-101(Fe) of **a** ATP and **b** EY (100 ppm)

The graph of ln (C_e_) vs. q_e_ (Fig. 10a and b) was used to calculate the Temkin isotherm constants, b_t_ and At. Table 1 shows the correlation coefficient and Temkin constant values. The Langmuir model fits the experimental data better than the Temkin model, as shown by the derived R^2^ values of 0.9812, and 0.93622 for the Langmuir model of ATP insecticide and EY dye on MIL-101(Fe) while on NH_2_-MIL-101(Fe) was 0.9795 and 0.9999, respectively. The R^2^ of Temkin model for ATP and EY adsorption on MIL-101(Fe) was 0.89136 and 0.91393 while on NH_2_-MIL-101(Fe) was 0.88805, and 0.87822 respectively.

### Adsorption kinetics on the MIL-101(Fe) and NH_2_-MIL-101(Fe) surface

Various kinetic models, including the pseudo-first-order, pseudo-second-order, Elvoich, Boyd kinetic, and intraparticle diffusion models, were employed to match the experimental data to examine the adsorption mechanism of ATP insecticide and EY dye onto MIL-101(Fe) and NH_2_-MIL-101(Fe).

#### Pseudo-first-order and second-order model

Lagergren model was applied to analyze the adsorption kinetics of MIL-101(Fe) and NH_2_-MIL-101(Fe) to better understand the link between the two compounds adsorption processes for the removal of ATP pesticide and EY dye (Liu et al. 2021). The adsorption rate constant may be determined by applying the 1st-order reaction kinetic model and pseudo-2nd-order kinetic model. The Lagergren first-order model linear form is illustrated as follows (Adly et al. 2021; Younes et al. 2021).

**Fig. 11:**
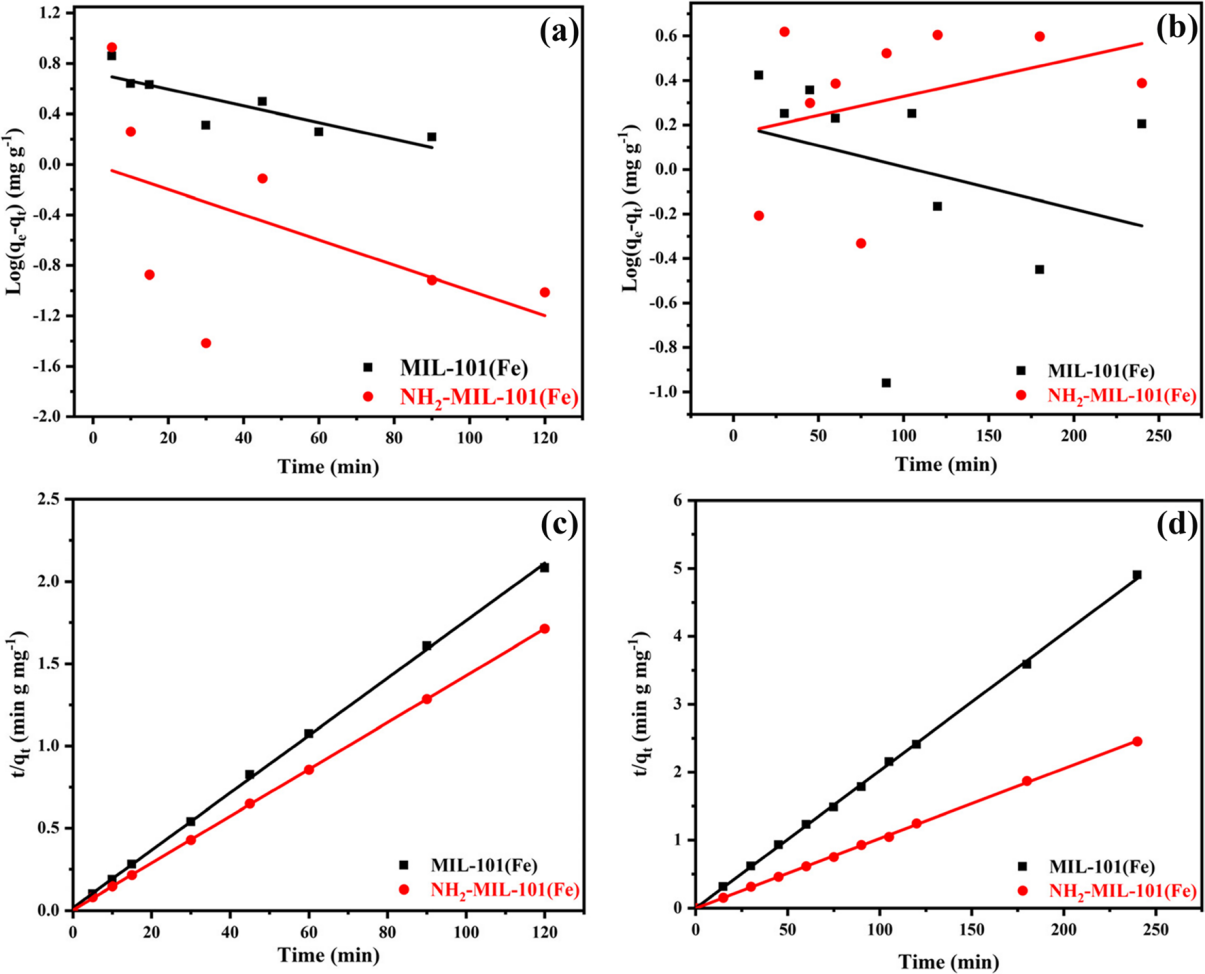
The pseudo-first and pseudo-second orders kinetic for the adsorption of ACT (**a**, **c**) and EY (**b** and **d**) on NH2-MIL-101 adsorbent

Pseudo-first order equation:

15 $$\mathrm{log}\left({\mathrm{q}}_{\mathrm{e}}- {\mathrm{q}}_{\mathrm{t}}\right)=\mathrm{ log}{\mathrm{q}}_{\mathrm{e}}- \frac{{\mathrm{k}}_{1}\mathrm{t}}{2.303}$$

where ${\mathrm{k}}_{1}$ (min^−1^) is the constant of rate for the 1st-order model, ${\mathrm{q}}_{\mathrm{e}}$ and ${\mathrm{q}}_{\mathrm{t}}$ (mg.g^−1^) are the quantities adsorbed at equilibrium and time $\mathrm{t}$ (min), correspondingly, when ${\mathrm{k}}_{1}$ is calculated from the t against $\mathrm{log}\left({\mathrm{q}}_{\mathrm{e}}-{\mathrm{q}}_{\mathrm{t}}\right)$ plots for every sample at various concentrations and temperatures, a line that is straight and a slope of ( ${\mathrm{k}}_{1}$/2.303) and an intercept of ${\mathrm{logq}}_{\mathrm{e}}$ is produced. The received information was assessed to see whether it was valid for either the Lagergren equation or pseudo-first order. The experimental findings ${\mathrm{q}}_{\mathrm{e}}$ as displayed in Table 2 and Fig. 11 are much greater than the estimated values. Because the model pseudo-1st order R^2^ values are lower than those of the model of the 2nd order, the adsorption of the ATP pesticide and EY dye does not follow the kinetics of pseudo-1st order when two various factors of concentration and temperatures.

**Table 2:** Kinetic parameters for the adsorption of ACT and EY on NH_2_-MIL-101 adsorbent

Model	Pollutant	ATP		EY dye	
	Adsorbent	MIL-101(Fe)	NH_2_-MIL-101(Fe)	MIL-101(Fe)	NH_2_-MIL-101 (Fe)
Pseudo-first order	${\mathrm{q}}_{\mathrm{e}}(\mathrm{mg}.{\mathrm{g}}^{-1})$	5.343	1.00	1.5993	1.4421
	${\mathrm{k}}_{1} ({\mathrm{min}}^{-1})$	0.01518	0.02303	0.004376	0.003915
	${\mathrm{R}}^{2}$	0.72917	0.27798	0.093	0.1260
Pseudo-second order	${\mathrm{q}}_{\mathrm{e}}(\mathrm{mg}.{\mathrm{g}}^{-1})$	57.471	70.422	49.504	97.090
	${\mathrm{k}}_{2} (\mathrm{g }{\mathrm{mg}}^{-1}\cdot {\mathrm{min}}^{-1})$	0.0175	0.0587	0.357	0.0290
	${\mathrm{R}}^{2}$	0.99947	0.99997	0.99953	0.99962
Elvoich	$\alpha \left(\mathrm{mg}.{\mathrm{g}}^{-1}{\mathrm{min}}^{-1}\right)$	6.20*10^10^	4.7268*10^13^	2.105*10^31^	3.114*10^90^
	$\upbeta (\mathrm{mg}.{\mathrm{g}}^{-1}{\mathrm{min}}^{-1/2})$	1.8889	1.9333	0.64789	0.48158
	${\mathrm{R}}^{2}$	0.89638	0.54127	0.33117	0.0658
Boyd kinetic	${\mathrm{B}}_{\mathrm{t}}$	2.104	7.06648	2.44843	4.58564
	${\mathrm{R}}^{2}$	0.62521	0.48904	0.24308	0.02631
Intraparticle Diffusion	$\mathrm{C }(\mathrm{mg}.{\mathrm{g}}^{-1})$	50.51594	65.16108	48.05752	98.94686
	${\mathrm{K}}_{\mathrm{d}} (\mathrm{mg}.{\mathrm{g}}^{-1} {\mathrm{min}}^{-0.5})$	0.65263	0.57409	0.12732	0.11086
	${\mathrm{R}}^{2}$	0.82597	0.3684	0.2312	0.06303

Equation of pseudo-second order:

16 $$\frac{\mathrm{t}}{{\mathrm{q}}_{\mathrm{t}}}= \frac{1}{{\mathrm{k}}_{2} {{\mathrm{q}}_{\mathrm{e}}}^{2}} + \frac{1}{{\mathrm{q}}_{\mathrm{e}}}\mathrm{t}$$

where ${\mathrm{k}}_{2}$ (g mg^−1^·min^−1^) is the model equilibrium rate constant for pseudo-2nd-order. The ATP pesticide and EY dye adsorbed amounts are ${\mathrm{q}}_{\mathrm{t}}$ and ${\mathrm{q}}_{\mathrm{e}}$, respectively, at equilibrium and at time t. Values of ${\mathrm{k}}_{2}$ and ${\mathrm{q}}_{\mathrm{e}}$ may be acquired by using the slope and intercept of plots t versus$\mathrm{t}/{\mathrm{q}}_{\mathrm{t}}$. The linear relationship between $\mathrm{t}$ and $\mathrm{t}/{\mathrm{q}}_{\mathrm{t}}$ demonstrates that chemisorption is the primary controlling stage in the adsorption process. The values obtained for R^2^ and the computed values of maximal adsorption capacity ${\mathrm{q}}_{\mathrm{e}}$ for the model of pseudo-2nd order are consistent with the experimental data and showed the use of the kinetic model of pseudo-2nd order (Fig. 11c and d). Correlation coefficients (R^2^) were 0.999 for the two Fe-based MOFs for the adsorption of ATP and EY dye from aqueous solutions. Furthermore, a capability for equilibrium adsorption predicted from appropriate findings was compatible with the experimental data of MIL-101(Fe) and NH_2_-MIL-101(Fe), which are given in Table 2. These results suggest that the model of pseudo-2nd-order, which was created depending on the hypothesis that a chemisorption process encompassing valency forces through the sharing (or exchange) of electrons among the adsorbate and the adsorbent might be the rate-limiting phase, is followed by the adsorption of pesticides and organic dyes on Fe-based MIL-101 (Li et al. 2020a; Liu et al. 2021; Xie et al. 2017).

The adsorption behavior of ATP pesticide and EY dye removal by MIL-101(Fe) and NH_2_-MIL-101(Fe) was described using pseudo-1st and 2nd-order kinetics. Table 2 and Fig. 11 display the main findings of the kinetics parameters. The main findings of the kinetics parameters are displayed in Fig. 11 and Table 2. The pseudo-2nd -order model (R^2^ = 0.99947, 0.99997, 0.99953, and 0.99962) describes the experimental data more accurately than the pseudo-1st-order model (R^2^ = 0.72917, 0.27798, 0.093 and 0.1260), according to the kinetics findings of the ATP insecticide and EY dye adsorption onto both MIL-101(Fe) and NH_2_-MIL-101(Fe). Additionally, the chi-square values and the good agreement between the computed and experimental q_e_ values support this. The pseudo-second model well fit of the ATP pesticide and EY dye adsorption on MIL-101(Fe) and NH_2_-MIL-101(Fe) demonstrating that chemisorption is the rate-limiting mechanism of the adsorption process and that the adsorption capacity is proportional to the active sites numbers onto adsorbents. Therefore, it was implied that ATP pesticide and EY dye were adsorbed onto the surface of MIL-101(Fe) and NH_2_-MIL-101(Fe) by chemical interactions, such as hydrogen bonding and the π–π EDA interaction (Isaeva et al. 2021; Li et al. 2023; Mahmoud et al. 2022).

#### Elovich model

**Fig. 12:**
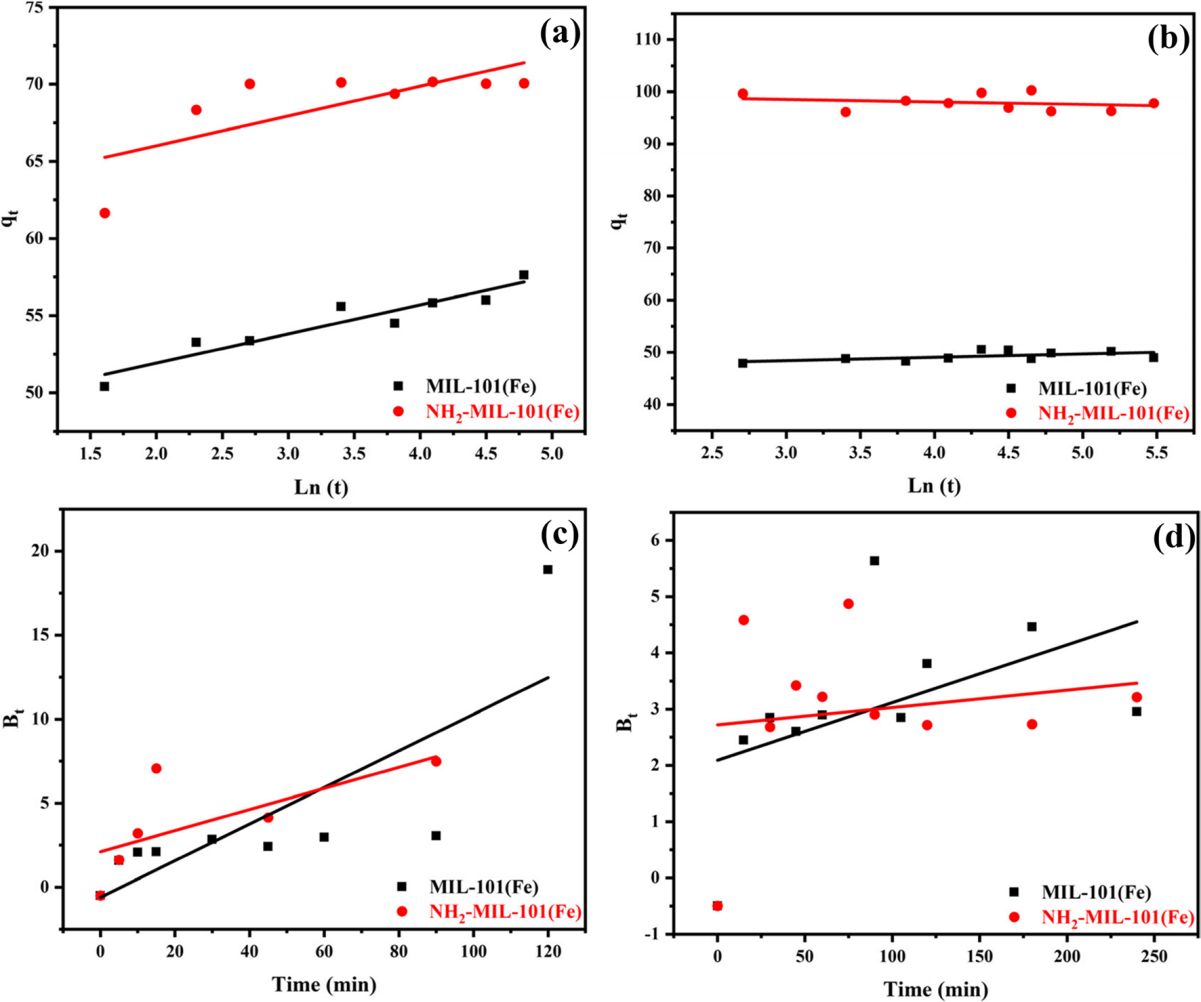
Elvoich and Boyd kinetic of MIL-101(Fe) and NH2-MIL-101(Fe) of **a** ATP and **b** EY (100 ppm)

The basic assumptions of the Elovich model are postulated as follows: (i) the activation energy grew as the adsorption period went on and (ii) the adsorbent surface was heterogeneous (Wang & Guo 2020b). In the presumption that the sorbent surface is energetically heterogeneous, this model is applied to clarify the pseudo-2nd-order kinetics (Yakub et al. 2020). As time goes on, the adsorption sites rise and multilayer. The Elovich model has also been used for the chemisorption of gases onto heterogeneous surfaces of adsorbent (Hoskins & Bray 1926). Elovich model describes the adsorption process as shown in Table 2 and Fig. 12 a and b by Eq. 18 as follows:

17 $${\mathrm q}_{\mathrm t}=\beta\mathrm{In}\left(a\beta\right)+\beta\left(\mathrm t\right)$$

where ${\mathrm{q}}_{\mathrm{t}}$ adsorbate concentration in the adsorbent at equilibrium (mg.g^−1^) and at a given contact time t ( min), α is the initial sorption rate (mg.g^−1^ min^−1^), and the parameter β (mg.g^−1^ min^−1/2^). It is related to the extent of surface coverage and activation energy for chemisorption. It can be calculated from the slopes and the intercepts of the linear plot between ln(t) versus q_t_ giving a straight line with the correlation coefficient 0.89638, 0.54127, 0.33117 and 0.0658 and β 1.8889, 1.9333, 0.64789, and 0.48158 (mg.g^−1^ min^−1/2^) (Benjelloun et al. 2021, Thabet & Ismaiel 2016).

#### Boyd kinetic model

It is a model that enables the differentiation of intraparticle diffusion from extra particle diffusion: The sorption is regulated by intraparticle diffusion if the plot of B_t_ against time is a straight line that passes through the origin; if not, the diffusion in the film (which is constrained by extra particular transport) controls the sorption (Okewale et al. 2013). This model is described by Eqs. 19 and 20. The results are shown in Fig. 12 c and d in addition to Table 2.

18 $$\mathrm{F}=\frac{{\mathrm{q}}_{\mathrm{t}}}{{\mathrm{q}}_{\mathrm{e}}}=\left(1-\frac{6}{{\uppi }^{2}}\right) \mathrm{exp}- {\mathrm{B}}_{\mathrm{t}}$$

19 $${\mathrm{B}}_{\mathrm{t}}= -0.4977-\mathrm{ ln}\left(1-\mathrm{F}\right)$$

where q_e_ is the amount of adsorbate on the adsorbent at equilibrium (mg.g^−1^), B_t_ is the Boyd constant calculated by Eq. (20)., and q_t_ is the amount of adsorbate in the adsorbent at time t (mg.g^−1^). Graphing B_t_ as a function of contact time t and noting the coefficient of determination R^2^ makes it easy to evaluate this isotherm.

Figure 12 c and d shows the linear plot between t^0.5^ vs. B_t_. The constants and R^2^ values are shown in Table 2. However, the obtained value of R^2^ for the Boyd kinetic model (0.62521, 0.48904, 0.24308 and 0.02631) was found to be less than that obtained for the other models.

#### Intraparticle diffusion model

**Fig. 13:**
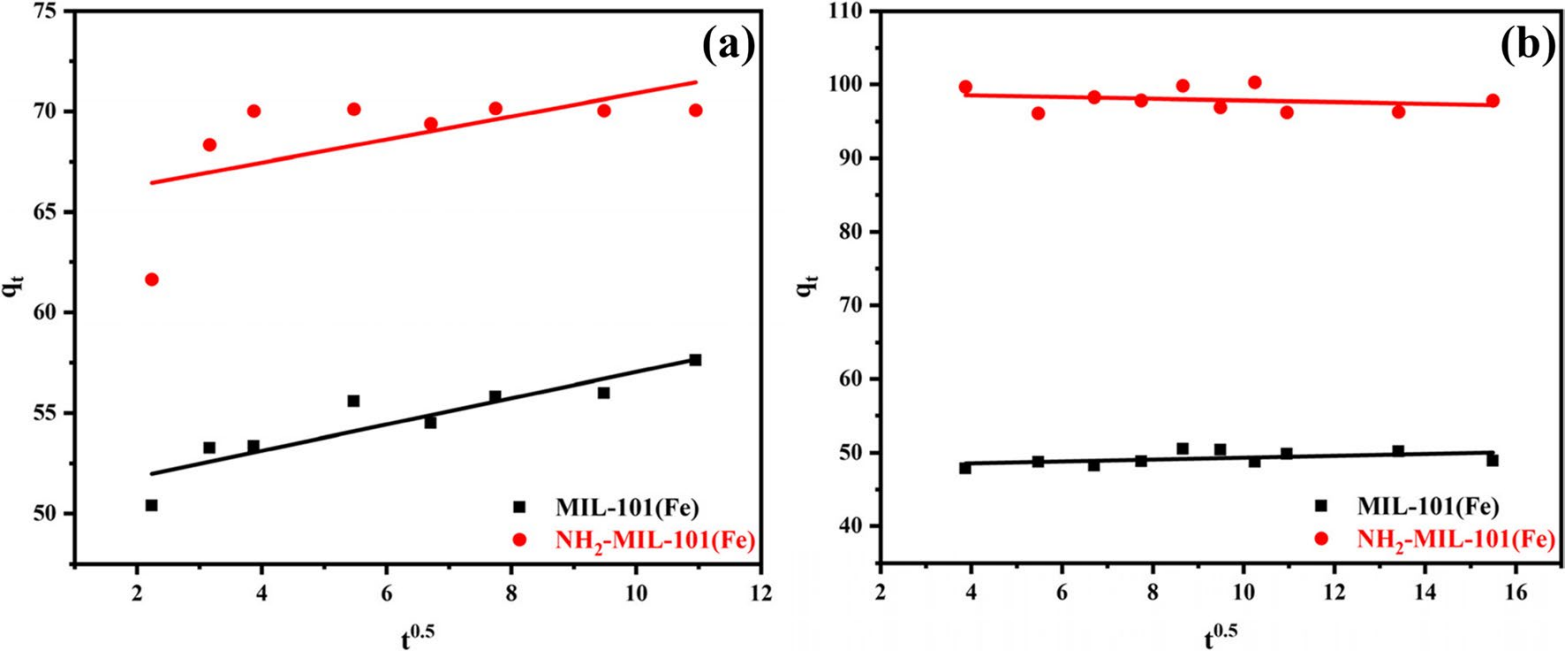
Intraparticle diffusion of MIL-101(Fe) and NH2-MIL-101(Fe) of **a** ATP and **b** EY (100 ppm)

It is possible to regulate the adsorption of ACT insecticide and EY dye onto NH_2_-MIL-101(Fe) adsorbent by either intraparticle diffusion, external film diffusion, or both (Mahanna & Azab 2020). The intraparticle diffusion model given in Eq. (21) is utilized to determine the diffusion process, and Fig. 13 shows this in addition to Table 2.

20 $${\mathrm{q}}_{\mathrm{t}}= {\mathrm{K}}_{\mathrm{d}}{\mathrm{t}}^{0.5}+\mathrm{C}$$

where k_d_ (mg.g^–1^ min^–0.5^) is the intraparticle diffusion rate constant, C (mg.g^–1^) is the intercept that indicates the boundary layer thickness and q_t_ (mg.g^–1^) is the adsorption capacity at time t (min).

Table 2 displays the constants and R^2^ values. These results verify that the adsorption of ATP insecticide and EY dye onto MIL-101(Fe) and NH_2_-MIL-101(Fe) adsorbent is significantly influenced by the intraparticle diffusion process. However, it was discovered that the intraparticle diffusion model's R^2^ value (0.82597, 0.3684, 0.2312, and 0.06303) was lower than the values obtained for the other models.

Results indicate that the pseudo-second-order model was able to match the experimental data more accurately than other models, according to the findings of the kinetic models of the adsorption of ATP pesticide and EY dye onto MIL-101(Fe) and NH_2_ MIL-101(Fe) adsorbent. The pseudo-2nd-order kinetics regression coefficient (R^2^) values are near unity (0.99947, 0.99997, 0.99953 and 0.99962). Different adsorption models have a lower correlation coefficient than other relevant models; the order is as follows: pseudo-2nd-order (0.99947, 0.99997, 0.99953, and 0.99962) > Elvoich model (0.89638, 0.54127, 0.33117 and 0.0658) > intraparticle diffusion (0.82597, 0.3684, 0.2312, and 0.06303) > pseudo-1st order (0.72917, 0.27798, 0.093 and 0.1260) > Boyd kinetic (0.62521, 0.48904, 0.24308, and 0.02631).

### Thermodynamic parameters

#### Van’t hoff equation

Whether the sorption process follows physisorption or chemisorption is determined by the organic pollutants’ thermodynamic behavior as they sorb from an aqueous solution onto the NH_2_-MIL-101(Fe) adsorbent (Sahmoune 2018). Various adsorption parameters were studied at various temperatures.

The formulas equations listed below were applied to calculate the free energy of Gibbs (∆G°), enthalpy (ΔH°), and entropy (ΔS°) using Van't Hoff equation:

21 $${\Delta \mathrm{G}}^{\mathrm{o}}={\Delta \mathrm{H}}^{\mathrm{o}}-\mathrm{T}{\Delta \mathrm{S}}^{\mathrm{o}}$$

22 $${\Delta \mathrm{G}}^{\mathrm{o}}=-\mathrm{RTln}\left(\uprho {\mathrm{K}}_{\mathrm{c}}\right)$$

23 $$\mathrm{ln}\left(\uprho {\mathrm{K}}_{\mathrm{c}}\right)=-\frac{{\Delta \mathrm{H}}^{\mathrm{o}}}{\mathrm{RT}}+\frac{{\Delta \mathrm{S}}^{\mathrm{o}}}{\mathrm{R}}$$

24 $${\Delta \mathrm{S}}^{\mathrm{o}}=\mathrm{intercept}\times \mathrm{R}$$

25 $${\mathrm{E}}_{a}=-\mathrm{slope}\times \mathrm{R}$$

where ρ (g.L^−1^) is the density of water, R is a constant of an ideal gas (8.3145 J.mol^−1^ K^−1^), and the temperature is T (K). K_C_ is the constant of equilibrium and was estimated as qe/Ce, while ΔH and ΔS were determined from the linear plot of 1/T against ln $(\uprho {\mathrm{K}}_{\mathrm{c}})$, including its slope and intercept Eq. 24. ΔG° is a change of Gibbs free energy, ΔH° is the enthalpy change, and ΔS° is the entropy change (Mohammad & El-Sayed 2020). Values of the equilibrium constant and q_e_/C_e_ are enumerated in Table 3. The negative ∆G° values reveal that the adsorption of the organic pollutants on nanoparticles is a spontaneous process for ACT pesticide and EY dye. Using the slope and intercept of the 1/T vs In (pK_c_). plot provided in Eqs. 22 and 23, we get the ΔH° and ΔS° values, in that order. If ∆H° is less than 20 kJ.mol^−1^, the adsorption is physisorption in nature and includes weak forces of attraction or Van der Waals forces, which are frequently thought of as outer-sphere complexation between the surface of the adsorbent and the dye the ∆S° negative value points that throughout the adsorption process, the state of orderliness at the solid/solution interface increased, and the adsorbate and adsorbents underwent some structural modifications.

**Table 3:** Thermodynamic parameters for the adsorption of ATP and EY dye onto NH_2_-MIL-101(Fe)

Pollutant			Van’t Hoff		
	T (K)	ΔG° (kJ.mol^−1^)	ΔS° (kJ^−1.^mol^−1^)	ΔH° (kJ.mol^−1^)	K
ATP	283 293 303 313	-20.39 -20.92 -21.42 -21.95	52.88	5.428	5.80 5.38 4.98 4.66
EY dye	298 308 318 328	-16.74 -17.20 -17.59 -18.04	57.16	2.012	0.86 0.82 0.78 0.75
		**Arrhenius**	**`**	**Modified Arrhenius**	
ATP	${\mathrm{E}}_{\mathrm{a}}(\mathrm{kJ}.{\mathrm{mol}}^{-1})$	5.428	${\mathrm{E}}_{\mathrm{a}}(\mathrm{kJ}.{\mathrm{mol}}^{-1})$	4.553	
EY dye		2.012		1.624	
ATP	$\mathrm{A}({\mathrm{dm}}^{3}.{\mathrm{mol}}^{-1}{\mathrm{S}}^{-1})$	10.74552	${\mathrm{S}}^{*}$	0.025	
EY dye		6.87577		0.29	
Bold refers to model name.					

#### Arrhenius equation

**Fig. 14:**
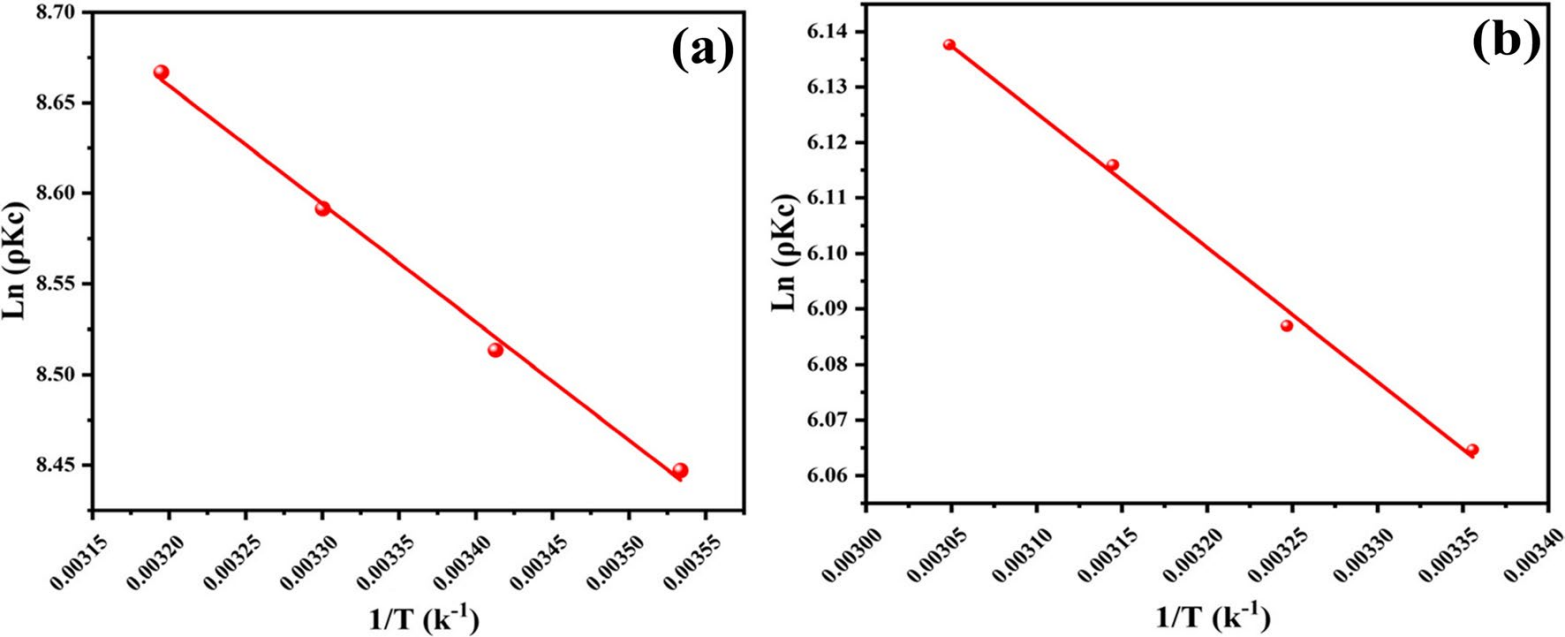
The Van’t Hoff equation of adsorption **a** ATP and **b** EY dye

**Fig. 15:**
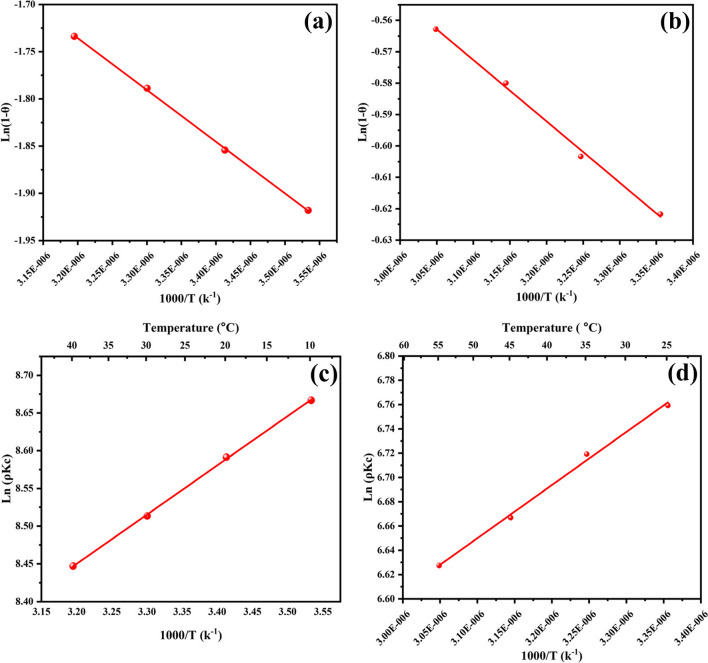
Arrhenius of adsorption **a** ATP and **b** EY dye. Modified Arrhenius of adsorption **c** ATP and **d** EY dye

When the equation of Arrhenius is applied to determine the energy of activation, the linear form is expressed as follows: it is possible to identify the kind of adsorption process since the rate of reaction rises with increased temperature as shown in Fig. 15a and b by Eqs. 27 and 28 as follows:

Activation energy:

26 $$\mathrm{In}\left(\uprho {\mathrm{K}}_{\mathrm{c}}\right)=\mathrm{InA}-\frac{{\mathrm{E}}_{\mathrm{a}}}{\mathrm{RT}}$$

27 $$\mathrm{Slope}=-\frac{{\mathrm{E}}_{\mathrm{a}}}{\mathrm{R}}$$

where A is a factor of pre-exponential and E_a_ is the energy of activation is calculated by Eg (28), and R is the constant of gas (8.31 kJ^−1^.mol^−1^). The value of E_a_ may be computed using the slope of the curve among 1/T and In (pK_c_) (Table 3, Fig. 14). From an equation of Arrhenius, the activation energy value is 5.428 kJ.mol^−1^ (ATP) and 2.012 kJ.mol^−1^ (EY) verified that the process of chemisorption based on the results which taken from the equation of Van’t Hoff. The intercept values were 10.74552 and 6.87577 dm^3^.mol^−1^ s^−1^ for ATP and EY dye, respectively.

#### Modified arrhenius equation (probability sticking)

As stated by the Arrhenius type equation modified, the activation energy (${\mathrm{E}}_{\mathrm{a}}$) from Table 3 and Fig. 15 c and d and the likelihood of sticking (${\mathrm{S}}^{*}$) from the surface covering (θ) may be calculated (31).

28 $$\mathrm{In}\frac{\uprho {\mathrm{K}}_{2}}{\uprho {\mathrm{K}}_{1}}=-\frac{{\mathrm{E}}_{\mathrm{a}}}{\mathrm{R}}{\left[\frac{1}{{\mathrm{T}}_{2}}+\frac{1}{{\mathrm{T}}_{1}}\right]}^{-1}$$

29 $${\mathrm{E}}_{\mathrm{a}}=-\mathrm{R In}\frac{\uprho {\mathrm{K}}_{2}}{\uprho {\mathrm{K}}_{1}}{\left[\frac{1}{{\mathrm{T}}_{2}}+\frac{1}{{\mathrm{T}}_{1}}\right]}^{-1}$$

30 $${\mathrm{S}}^{*}=\left(1-\uptheta \right){\mathrm{e}}^{-{\mathrm{E}}_{\mathrm{a}}/\mathrm{RT}}$$

The ${\mathrm{S}}^{*}$ is considered a function of the adsorbate/adsorbent system that is the subject of the inquiry; it has a value that ranges from 0 < ${\mathrm{S}}^{*}$  < 1 and is influenced via the system's temperature. The value of θ may be obtained using the equation that follows.

31 $$\uptheta = \left[1-{\mathrm{C}}_{\mathrm{e}}/{\mathrm{C}}_{\mathrm{o}}\right]$$

The slope of the 1/T vs. ln (1 − θ) plot was used to determine the value of ${\mathrm{E}}_{\mathrm{a}}$. The size of the activation energy provides insight into the primary physical or chemical nature of the adsorption. Chemisorption is implied via higher activation energies (40–800 kJ.mol^–1^), whereas activation energies for physisorption processes usually lie between 5 and 40 kJ mol^−1^. The positive value of ${\mathrm{E}}_{\mathrm{a}}$ 4.553 (ATP) and 1.624 (EY) kJ.mol^−1^ showed that adsorption is a physisorption process that is exothermic in nature and prefers lower temperatures; the value of ${\mathrm{S}}^{*}$ is < 1, (0.025) (ATP) and (0.29) (EY) thus the sticking probability of acetamiprid and eosin Y uptake on the NH_2_-MIL-101(Fe) is very high.

### Mechanism of MIL-101(Fe) and NH_2_-MIL-101(Fe) adsorbent

**Fig. 16:**
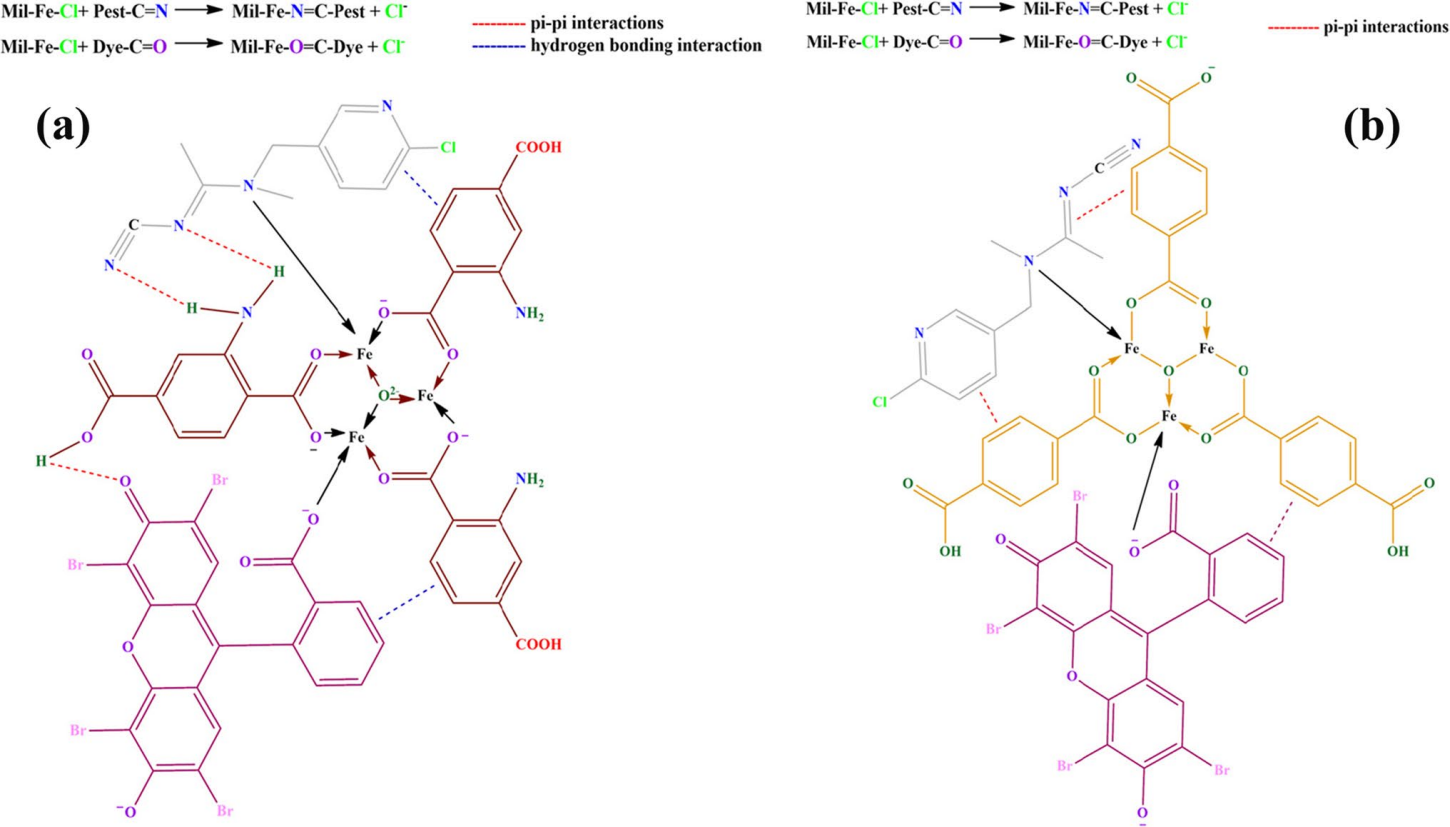
Mechanism of MIL-101(Fe) adsorbent with ATP pesticide and EY dye (**a**), NH2-MIL-101(Fe) adsorbent with ATP pesticide and EY dye (**b**)

Possible adsorption mechanisms for dye and pesticide are depicted in Fig. 16 which shows that it might be portrayed to π-π stacking, hydrogen bonding, and inner sphere coordination (Isaeva et al. 2021; Li et al. 2023; Mahmoud et al. 2022, Mondol & Jhung 2021).

Because of their versatile complexing activity towards a wide range of metal ions, carboxylates are a family of ligands for the synthesis of coordination compounds that have received much research. Because it is feasible to add extra groups to the ring to provide beneficial characteristics and interactions for desired applications, benzene carboxylic acids have been a particularly wise option. Hydrogen bonding interactions can be conferred by functionalities including thio, hydroxyl, nitro, amino, and halogens by serving as hydrogen donors or acceptors (Aris et al. 2020; Lunardi et al. 2022). The aromaticity of the benzene ring can also result in π-π interactions along with other aromatics and be helpful for adsorptive separation. In contrast to MIL-101(Fe), NH_2_—MIL-101(Fe) exhibits structural variety because the amine group is added together with carboxylate ligands (Bagheri et al. 2021; Xu et al. 2021). The dye molecule’s aromatic structure is responsible for powerful interactions with the frameworks including electrostatic interactions, π-π interaction, and physical adsorption (Mondol & Jhung 2021).

## Conclusion

Within this research, MIL-101(Fe) and NH_2_-MIL-101(Fe) nanocomposites were made up, and their capacities to extract ATP insecticide and EY dye from an aqueous solution were assessed. High adsorption capacities 57.6 and 48.9 mg/g of MIL-101(Fe) and 70.5 and 97.8 mg/g of NH_2_-MIL-101(Fe) for ATP and EY dye, respectively. To ensure that determine the removal effectiveness, the influences of contact duration, pH, the concentration of pollutants, and adsorbent dose were studied. The optimum contact duration by both adsorbents was discovered to be 10 min and 15 min for ATP pesticide and EY dye, correspondingly. The isotherm of both adsorbents matched well with the Langmuir model. The pseudo-2nd order model describes the adsorption kinetics rather well. The thermodynamic results show exothermic and non-spontaneous, and there is an increase in the state of orderness in the adsorption processes. According to a local energy decomposition study, electrostatic interactions are the main adsorption mechanism by which MIL-101(Fe) and NH_2_-MIL-101(Fe) remove pesticides and dyes. In summary, this work shows that MIL-101(Fe) and NH_2_-MIL-101(Fe) are efficient in eliminating dyes and pesticides from aqueous solutions.

### Supplementary Information

Below is the link to the electronic supplementary material.

Supplementary file1 (DOCX 6160 KB)

## Data Availability

The experimental data of this study is available upon request
