# Supplementary material for: Effective removal of acetamiprid and eosin Y by adsorption on pristine and modified MIL-101(Fe)
Source: Environ Sci Pollut Res Int. 2024 Jun 7;31(28):41221–45. doi: 10.1007/s11356-024-33821-w (PMC11190010; doi:10.1007/s11356-024-33821-w)
Supplement: Supplementary file 1 — Supplementary file1 (DOCX 6160 KB) [file 11356_2024_33821_MOESM1_ESM.docx]

**Effective removal of acetamiprid and Eosin Y by adsorption on pristine and modified MIL-101(Fe)**

Mohamed Sakr^a^, Mina Shawky^b^, Mohamed Gar Alalm^a^, Hani Mahanna^a^

^a^ Public Works Engineering Department, Faculty of Engineering, Mansoura University, Mansoura, 35516, Egypt

^b^ Chemistry Department, Faculty of Science, Mansoura University, Mansoura, 35516, Egypt

Supplementary Information

Fig. S1. Chemical structure of Acetamiprid (a) and Eosin Y (b).

**(a)**


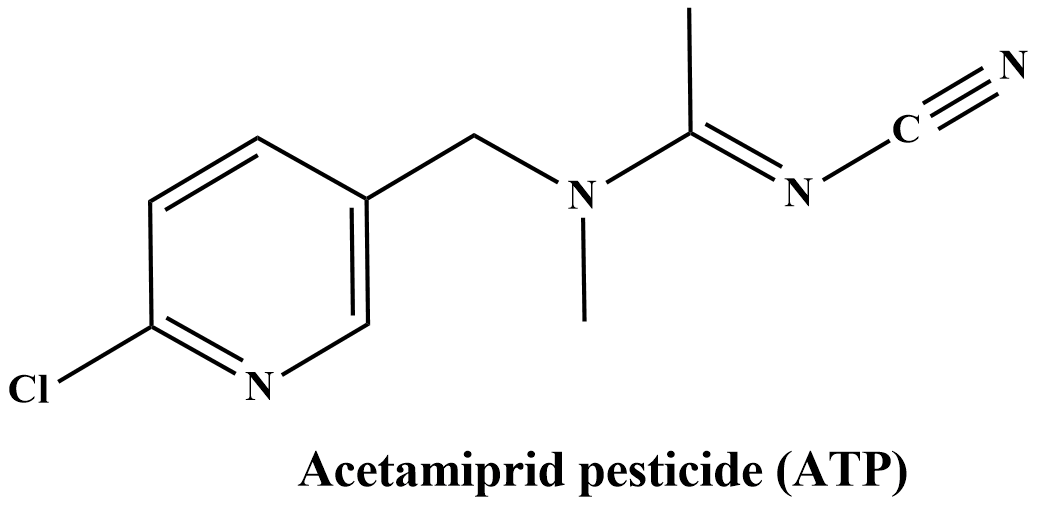

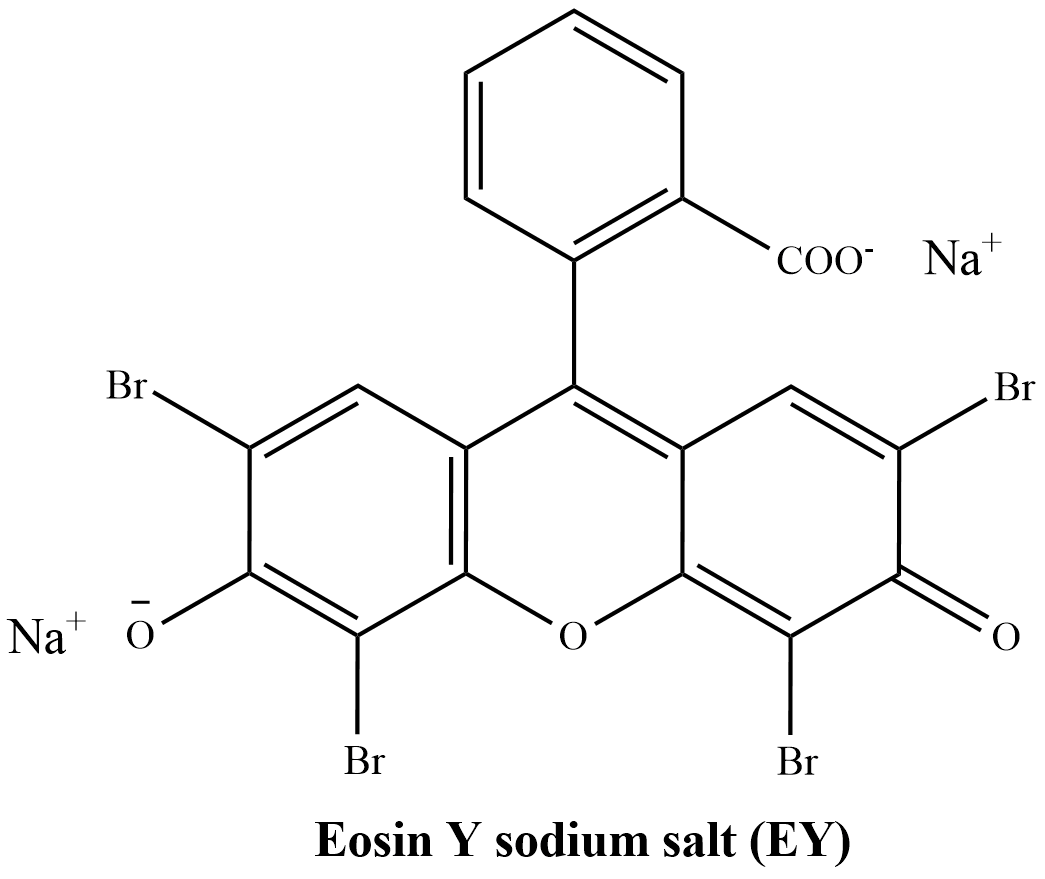


**(b)**

Fig. S2. Effect of Different MILs on the uptake of (a) ATP and (b) EY (10 & 5 ppm).

Fig. S3. Effect of Different MILs on the uptake of (a) ATP and (b) EY (50 ppm).

Fig. S4. Effect of Different MILs on the uptake of (a) ATP and (b) EY (100 ppm).

Fig. S5. Effect of Initial Concentration on the uptake of (a) ATP and (b) EY.

Fig. S6. Effect of Adsorbent Dosage on the adsorption capacities of (a) ATP and (b) EY.

Fig. S7. Effect of temperature on the adsorption capacities of (a) ATP and (b) EY.

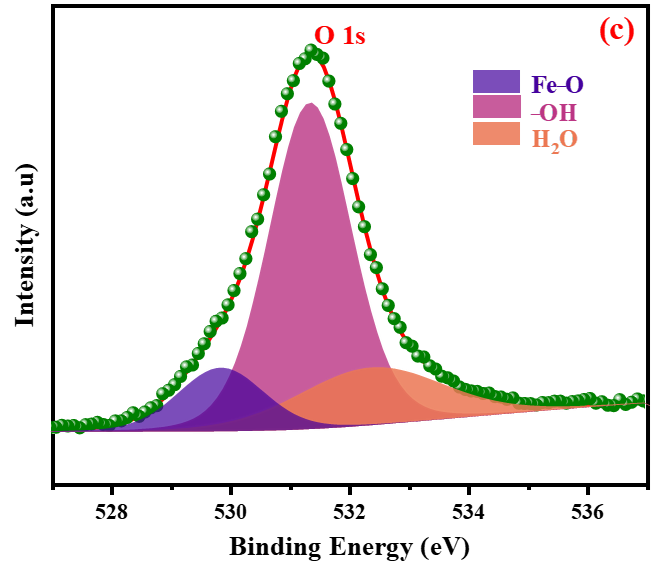


Fig. S8. (a) Full survey XPS of MIL-101(Fe) and HR-XPS of (b) C1s, (c) O 1s, (d) N 1s and (e) Fe 2p.


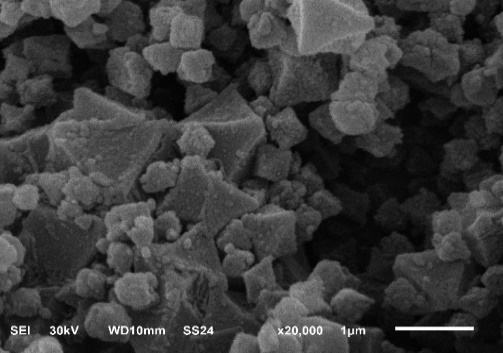

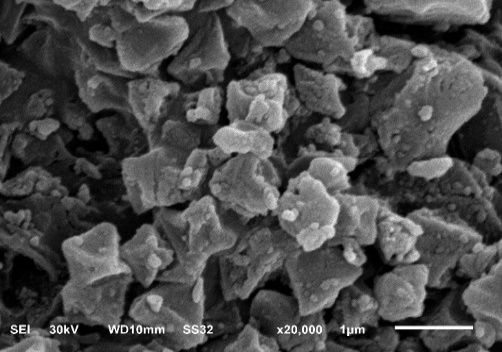

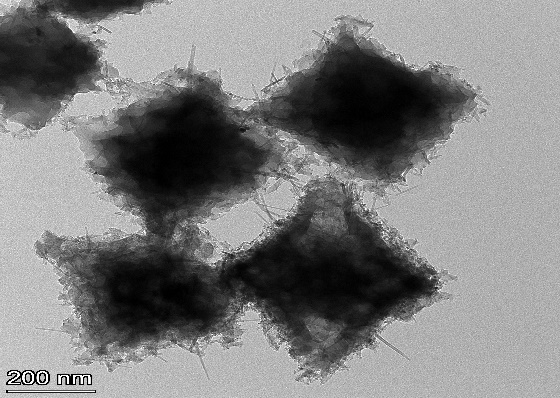

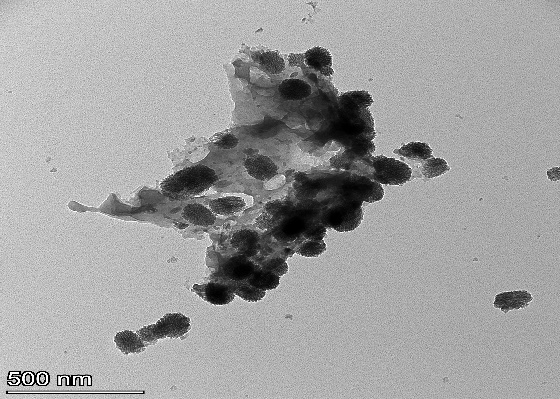


**(c)**

**(d)**

**(b)**

**(a)**

Fig. S 9. SEM images of (a) MIL-101(Fe) and (b) NH_2_-MIL-101(Fe) & TEM images of (c) MIL-101(Fe) and (d) NH_2_-MIL-101(Fe).


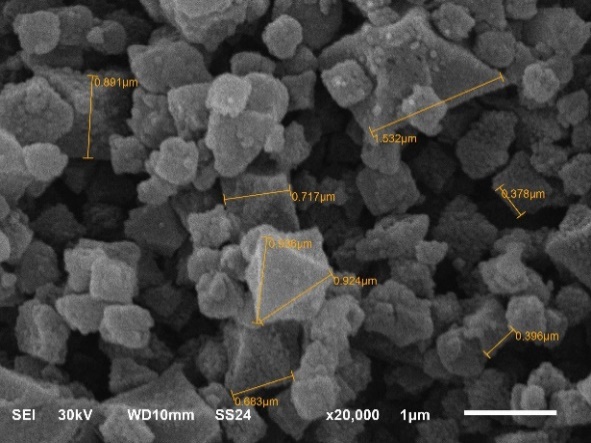

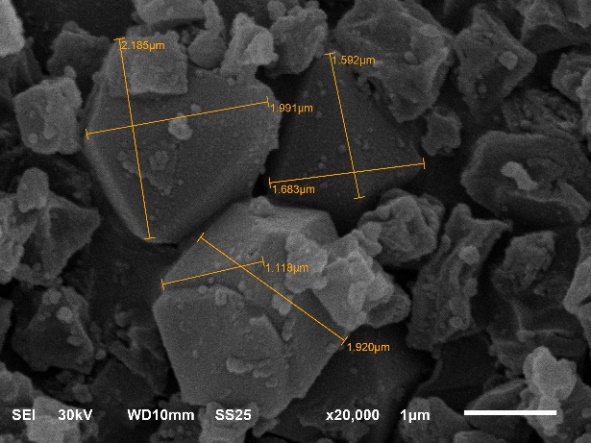

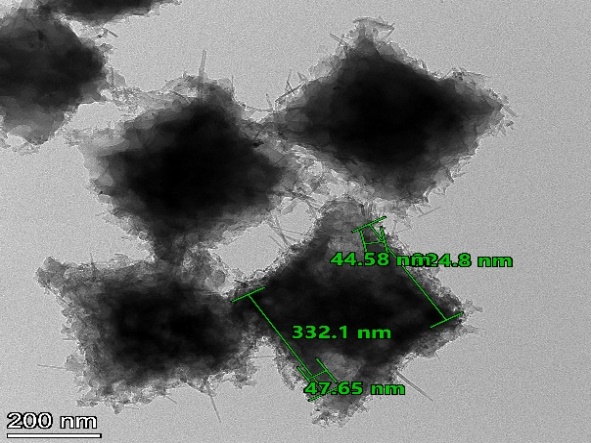

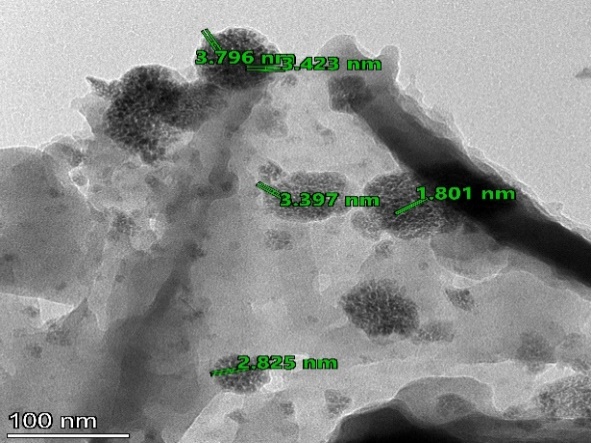


**(c)**

**(d)**

**(b)**

**(a)**

**(a)**

Fig. S10. Particels Size based on SEM images of (a) MIL-101(Fe) and (b) NH_2_-MIL-101(Fe) & TEM images of (c) MIL-101(Fe) and (d) NH_2_-MIL-101(Fe).

| Elem... | Weight% | Atomic% |
| --- | --- | --- |
| C K | 74.06 | 81.99 |
| O K | 19.94 | 16.58 |
| Fe K | 6.00 | 1.43 |


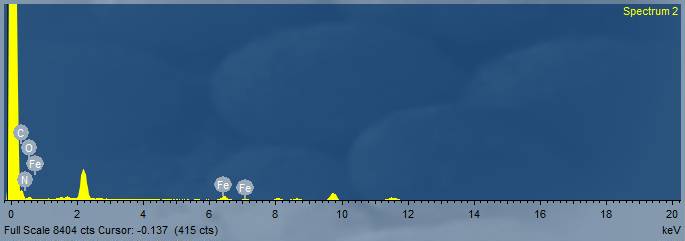

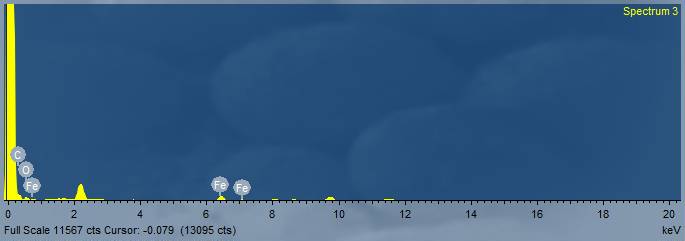


| Elem... | Weight% | Atomic% |
| --- | --- | --- |
| C K | 31.68 | 36.52 |
| N K | 41.87 | 41.40 |
| O K | 25.15 | 21.76 |
| Fe K | 1.30 | 0.32 |

Fig. S11. EDX images of (a) MIL-101(Fe) and (b) NH_2_-MIL-101(Fe).


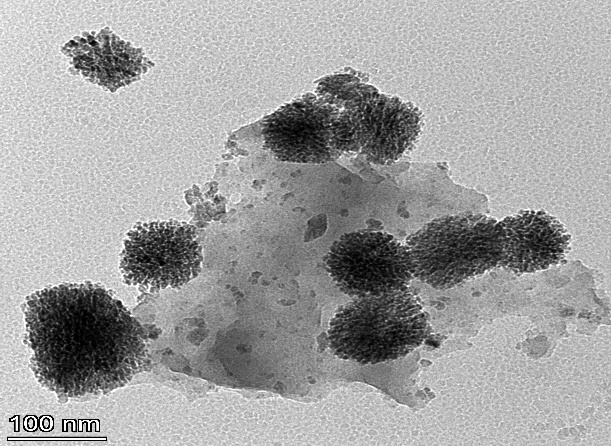

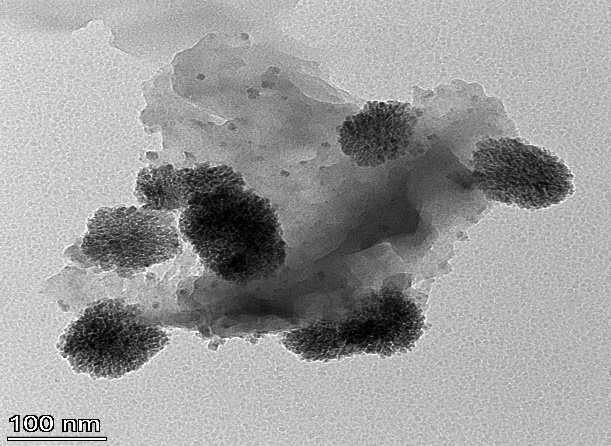

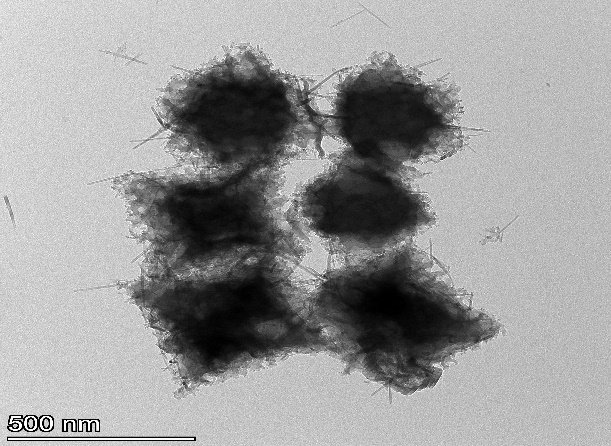

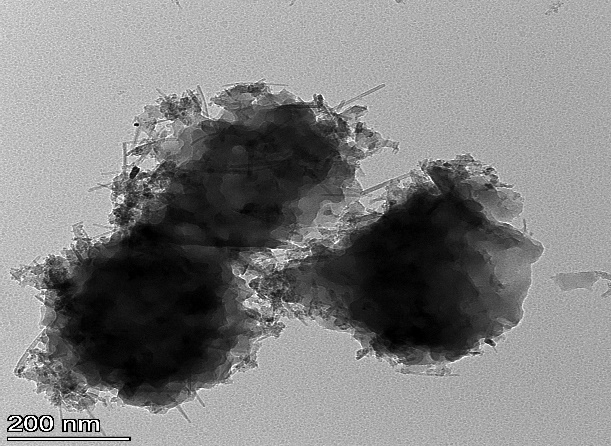


**(c)**

**(d)**

**(b)**

**(a)**

Fig. S12. TEM images of NH_2_-MIL-101(Fe) After Adsortption of (a) ATP pesticide & (b) EY dye.

Fig. S13. XRD of NH_2_-MIL-101(Fe) After Adsortption of (a) ATP pesticide & (b) EY dye.

Table S1 Crystallite Size from XRD of (a) MIL-101(Fe) and (b) NH_2_-MIL-101(Fe).

| Crystallite Size | | | | | | | |
| --- | --- | --- | --- | --- | --- | --- | --- |
| (a) MIL-101(Fe) | | | | **(b) NH_2_-MIL-101(Fe)** | | | |
| 2-theta | **FWHM** | **D(nm)** | **D(µm)** | **2-theta** | **FWHM** | **D(nm)** | **D(µm)** |
| 9.49223 | 0.60108 | 13.26217 | 0.013262 | 18.63106 | 0.12263 | 65.64837 | 0.065648 |
| 4.3781 | 4.75907 | 1.670514 | 0.001671 | 42.36745 | 4.11383 | 2.071067 | 0.002071 |
| 12.05027 | 13.19427 | 0.605446 | 0.000605 | 9.11277 | 1.25661 | 6.342044 | 0.006342 |
| 16.66428 | 0.22399 | 35.84552 | 0.035846 | 9.11277 | 44.88774 | 0.177542 | 0.000178 |
| 18.92191 | 1.00258 | 8.033113 | 0.008033 | 16.55607 | 0.31621 | 25.38797 | 0.025388 |
| 19.10699 | 10.39213 | 0.775204 | 0.000775 | 33.15976 | 2.57977 | 3.213046 | 0.003213 |
| 26.05369 | 52406.15 | 0.000156 | 1.56E-07 | 24.75189 | 51.01351 | 0.159434 | 0.000159 |
| 42.61568 | 3.33957 | 2.553381 | 0.002553 | 24.75189 | 22.18416 | 0.366626 | 0.000367 |
| 26.05369 | 32.47254 | 0.251109 | 0.000251 | 28.50943 | 1.50382 | 5.450558 | 0.005451 |

Table S2 Studying Pollutant Adsorption Catalytically Using the Adsorption Principle

| **Pollutants** | **Catalyst** | | **Approach** | | **Experimental parameters** | | **Time (min)** | **Max. capacity, q_e_ (mg.g^−1^)** | | **Ref.** | |  |
| --- | --- | --- | --- | --- | --- | --- | --- | --- | --- | --- | --- | --- |
| Metolachlor Alachlor  Acetochlor Pretilachlor | β-CD MOF-NPC | | Adsorption  processe | | 300 mL solution with 50 mg L^−1^ herbicides and 1 g  L^−1^ of absorbent, pH: 2 | | 450 | 343.42  291.26  261.21  311.78 | | **^(Liu et al. 2019)^** | |  |
| Bentazon, Clopyralid Isoproturon | MOF-235 (Fe) | | Adsorption  processe | | Conc.: 10 mg L^−1^, , pH:7 | | 60 | 10.00  9.76  7.15 | | **^(De Smedt et al. 2015)^** | |  |
| Neonicotinoid insecticides (thiamethoxam, imidacloprid, acetamiprid, nitenpyram, dinotefuran, clothianidin, and thiacloprid) | M−MOF | | Adsorption  processe | | Conc.: 50 mg.L^−1^, dose : 5 mg , pH: 7.4 | | 100 | 0.362 | | **^(Liu et al. 2017)^** | |  |
| Neonicotinoid insecticides | Co-MNPC | | Adsorption  processe | | Conc.: 40 mg.mL^−1^, , dose : 7.5 mg  pH: 6 | | 20 | - | | **^(Hao et al. 2014)^** | |  |
| imidacloprid | NH_2_-Fe-MILs | | Adsorption and Fenton  - like processes | | 40 mg/L IMC; 0.24 g/L NH_2_-MIL101(Fe) or 0.3 g/L NH_2_-MIL88B(Fe); 2.0 μL/mL H_2_O_2_; pH 6.4; | | 150 | - | | **^(Chen et al. 2021)^** | |  |
| glyphosate | MIL-101(Fe) ,  NH_2_-MIL-101(Fe) | | Adsorption  processe | | A 5 × 10^-4^ M glyphosate solution was prepared in water (40 mL),  dose : 5 mg,  pH: 6 | | 420 | 138.491  243.224 | | **^(Liu et al. 2022)^** | |  |
| ATP | MIL-101(Fe) | | Adsorption  processe | | Conc.: 100 mg.mL^−1^, dose : 5 mg,  pH: 6 | | 120 | 57.63 | | **^This study^** | |  |
| ATP | NH_2_-MIL-101(Fe) | | Adsorption  processe | | Conc.: 100 mg.mL^−1^, dose : 5 mg,  pH: 4 | | 120 | 70.51 | | **^This study^** | |  |
| Methyl Orange | GO-MIL-101(Fe)- | | Adsorption  processe | | Conc.: 100 mg.mL^−1^, dose :50 mg,  pH = 3–4 | | 180 | 186.20 | | **^(Liu et al. 2021b)^** | |  |
| Rhodamine B | MIL-53(Fe)/MBC | | Adsorption  processe | | Conc.: 5-1000 mg.mL^−1^,  dose : 50 mg,  pH = 6, | | 180 | 55 | | **^(Navarathna et al. 2019)^** | |  |
| methylene  blue | MIL-101(Fe) | | Adsorption, photocatalytic processes | | Conc.: 10 mg.mL^−1^,  dose : 5 mg,  pH: 10 | | 90 | 576.13 | | **^(Fattahi et al. 2023)^** | |  |
| Eosin Y | AC-MOF-5 | Adsorption  processe | | Conc.: 6 mg.mL^−1^,  dose : 3 mg,  pH: 7 | | - | | | 20.24 | | **^(Askari et al. 2017)^** | |
| EY | MIL-101(Fe) | Adsorption  processe | | Conc.: 100 mg.mL^−1^, dose : 5 mg,  pH: 7 | | 240 | | | 48.92 | | **^This study^** | |
| EY | NH_2_-MIL-101(Fe) | Adsorption  processe | | Conc.: 100 mg.mL^−1^, dose : 5 mg,  pH: 7 | | 240 | | | 97.82 | | **^This study^** | |

Table S3 Comparison of adsorption capacities of ACT by different adsorbents.

| **Number** | **Adsorbent** | $\mathbf{q}_{\mathbf{e}}\mathbf{(mg}\mathbf{g}^{\mathbf{-1}}\mathbf{)}$ | **Ref.** |
| --- | --- | --- | --- |
|  | **Bentonite** | **9.17** | **^(Choumane &Benguella 2016)^** |
|  | **Kaolin** | **7.7** | **^(Choumane &Benguella 2016)^** |
|  | **Tangerine peels activated carbon** | **35.7** | **^(Mohammad et al. 2020)^** |
|  | **Eucalyptus wood** | **4.78** | **^(Srikhaow et al. 2022)^** |
|  | **AC** | **10.85** | **^(Nejadshafiee &Islami 2020)^** |
|  | **AC-Fe(NO_3_)_3_** | **60.6** | **^(Sanz-Santos et al. 2021)^** |
|  | **AC-Fe_2_(SO4)_3_** | **60.6** | **^(Sanz-Santos et al. 2021)^** |
|  | **Biochar (BC)** | **4.87** | **^(Srikhaow et al. 2022)^** |
|  | **MOF-Fe_4_O_3_–GO–β-cyclodextrin Magnetic nanocomposite (M− MOF)** | **2.96** | **^(Mondol &Jhung 2021)^** |
|  | **Mesoporous activated carbon from starch (ACS)** | **66.2** | **^(Suo et al. 2019)^** |
|  | **Activated carbon from waste hemp** | **12.20** | **^(Vukčević et al. 2015)^** |
|  | **Chestnut shells** | **4.6984** | **^(Cobas et al. 2016)^** |
|  | **Phenyl-modified magnetic graphene/ mesoporous silica** | **5.108** | **^(Wang et al. 2017)^** |
|  | **Magnetic graphene oxide–cyclodextrin** | **0.362** | **^(Liu et al. 2017)^** |
|  | **molecularly imprinted polymers**  **(MIP)** | **22.99** | **^(Wang et al. 2020)^** |
|  | **NH_2_-Fe-MILs** | **138.491**  **243.224** | **(Liu et al. 2022)** |
|  | **NH_2_-MIL-101(Fe)** | **-** | **^(Chen et al. 2021)^** |
|  | **MIL-101(Fe)** | **57.63** | **^This study^** |
|  | **NH_2_-MIL-101(Fe)** | **70.51** | **^This study^** |

Table S4 Comparison of adsorption capacities of different adsorbents for EY.

| **Number** | **Adsorbent** | $\mathbf{q}_{\mathbf{e}}\mathbf{(mg}\mathbf{g}^{\mathbf{-1}}\mathbf{)}$ | **Ref.** |
| --- | --- | --- | --- |
|  | **Clay/Carbon Composite (SBE/C)** | **11.15** | **^(Liu et al. 2021a)^** |
|  | **xGnP® graphite nanoplatelets** | **66.00** | **^(de Oliveira et al. 2019)^** |
|  | **Sol-gel γ-Al_2_O_3_ nanoparticles** | **47.78** | **^(Thabet &Ismaiel 2016)^** |
|  | **CoAl-LDH** | **41.52** | **^(Nazir et al. 2022)^** |
|  | **NiAl-LDH** | **60.67** | **^(Nazir et al. 2022)^** |
|  | **NiCoAl-LDH** | **78.74** | **^(Nazir et al. 2022)^** |
|  | **Nickel Oxide nanoparticles** | **5.2167** | **^(Jabbar et al. 2022)^** |
|  | **Activated carbon** | **86.78** | **^(Alwi et al. 2020)^** |
|  | **Functionalized silica** | **13.84** | **^(Dada et al. 2020)^** |
|  | **Chitosan hydrobeads** | **76** | **^(Zhang et al. 2020)^** |
|  | **CuO nanoparticles loaded on activated carbon** | **96.22** | **^(Dashamiri et al. 2016)^** |
|  | **ZnO nanorod loaded on activated carbon** | **93.46** | **^(Dil et al. 2016)^** |
|  | **AC-MOF-5** | **20.24** | **^(Askari et al. 2017)^** |
|  | **Monolithic HKUST-1** | **72.46** | **^(Parsazadeh et al. 2018)^** |
|  | **MIL-101(Fe)** | **48.92** | **^This study^** |
|  | **NH_2_-MIL-101(Fe)** | **97.82** | **^This study^** |

**References**

Alwi RS, Gopinathan R, Bhowal A, Garlapati C (2020) Adsorption Characteristics of Activated Carbon for the Reclamation of Eosin Y and Indigo Carmine Colored Effluents and New Isotherm Model. Molecules. <https://doi.org/10.3390/molecules25246014>.

Askari H, Ghaedi M, Dashtian K, Azghandi MHA (2017) Rapid and high-capacity ultrasonic assisted adsorption of ternary toxic anionic dyes onto MOF-5-activated carbon: Artificial neural networks, partial least squares, desirability function and isotherm and kinetic study. Ultrason. Sonochem. <https://doi.org/10.1016/j.ultsonch.2016.10.029>.

Chen M-L, Lu T-H, Long L-L, Xu Z, Ding L, Cheng Y-H (2021) NH_2_-Fe-MILs for effective adsorption and Fenton-like degradation of imidacloprid: Removal performance and mechanism investigation. Environmental Engineering Research. <https://doi.org/10.4491/eer.2020.702>.

Choumane FZ, Benguella B (2016) Removal of acetamiprid from aqueous solutions with low-cost sorbents. Desalination and Water Treatment. <https://doi.org/10.1080/19443994.2014.966332>.

Cobas M, Meijide J, Sanromán MA, Pazos M (2016) Chestnut shells to mitigate pesticide contamination. J Taiwan Inst Chem Eng <https://doi.org/10.1016/j.jtice.2015.11.026>.

Dada AO, Adekola FA, Odebunmi EO, Dada FE, Bello OM, Akinyemi BA, Bello OS, Umukoro OG (2020) Sustainable and low-cost Ocimum gratissimum for biosorption of indigo carmine dye: kinetics, isotherm, and thermodynamic studies. Int. J. Phytorem. <https://doi.org/10.1080/15226514.2020.1785389>.

Dashamiri S, Ghaedi M, Dashtian K, Rahimi MR, Goudarzi A, Jannesar R (2016) Ultrasonic enhancement of the simultaneous removal of quaternary toxic organic dyes by CuO nanoparticles loaded on activated carbon: Central composite design, kinetic and isotherm study. Ultrason. Sonochem. <https://doi.org/10.1016/j.ultsonch.2016.02.008>.

de Oliveira EHC, Fraga DMDSM, da Silva MP, Fraga TJM, Carvalho MN, de Luna Freire EMP, Ghislandi MG, da Motta Sobrinho MA (2019) Removal of toxic dyes from aqueous solution by adsorption onto highly recyclable xGnP® graphite nanoplatelets. Journal of Environmental Chemical Engineering. <https://doi.org/10.1016/j.jece.2019.103001>.

De Smedt C, Spanoghe P, Biswas S, Leus K, Van Der Voort P (2015) Comparison of different solid adsorbents for the removal of mobile pesticides from aqueous solutions. Adsorption. <https://doi.org/10.1007/s10450-015-9666-8https://doi.org/10.1007/s10450-015-9666-8>.

Dil EA, Ghaedi M, Ghaedi AM, Asfaram A, Goudarzi A, Hajati S, Soylak M, Agarwal S, Gupta VK (2016) Modeling of quaternary dyes adsorption onto ZnO–NR–AC artificial neural network: Analysis by derivative spectrophotometry. Journal of Industrial and Engineering Chemistry. <https://doi.org/10.1016/j.jiec.2015.11.010>.

Fattahi M, Niazi Z, Esmaeili F, Mohammadi AA, Shams M, Nguyen Le B (2023) Boosting the adsorptive and photocatalytic performance of MIL-101(Fe) against methylene blue dye through a thermal post-synthesis modification. Sci Rep. <https://doi.org/10.1038/s41598-023-41451-4>.

Hao L, Wang C, Wu Q, Li Z, Zang X, Wang Z (2014) Metal-organic framework derived magnetic nanoporous carbon: novel adsorbent for magnetic solid-phase extraction. Anal. Chem. <https://doi.org/10.1021/ac5031896>.

Jabbar NM, Salman SD, Rashid IM, Mahdi YS (2022) Removal of an anionic Eosin dye from aqueous solution using modified activated carbon prepared from date palm fronds. Chemical Data Collections. <https://doi.org/10.1016/j.cdc.2022.100965>.

Liu C, Wang P, Liu X, Yi X, Zhou Z, Liu D (2019) Multifunctional β-Cyclodextrin MOF-Derived Porous Carbon as Efficient Herbicides Adsorbent and Potassium Fertilizer. ACS Sustainable Chemistry & Engineering. <https://doi.org/10.1021/acssuschemeng.9b01911>.

Liu G, Li L, Xu D, Huang X, Xu X, Zheng S, Zhang Y, Lin H (2017) Metal–organic framework preparation using magnetic graphene oxide–β-cyclodextrin for neonicotinoid pesticide adsorption and removal. Carbohydr. Polym. <https://doi.org/10.1016/j.carbpol.2017.06.074>.

Liu R, Xie Y, Cui K, Xie J, Zhang Y, Huang Y (2022) Adsorption behavior and adsorption mechanism of glyphosate in water by amino-MIL-101(Fe). J. Phys. Chem. Solids. <https://doi.org/10.1016/j.jpcs.2021.110403>.

Liu Y, Chen Y, Shi Y, Wan D, Chen J, Xiao S (2021a) Adsorption of toxic dye Eosin Y from aqueous solution by clay/carbon composite derived from spent bleaching earth. Water Environ. Res. <https://doi.org/10.1002/wer.1376>.

Liu Z, He W, Zhang Q, Shapour H, Bakhtari MF (2021b) Preparation of a GO/MIL-101(Fe) Composite for the Removal of Methyl Orange from Aqueous Solution. ACS Omega. <https://doi.org/10.1021/acsomega.0c05091>.

Mohammad SG, Ahmed SM, Amr AEE, Kamel AH (2020) Porous Activated Carbon from Lignocellulosic Agricultural Waste for the Removal of Acetampirid Pesticide from Aqueous Solutions. Molecules. <https://doi.org/10.3390/molecules25102339>.

Mondol MMH, Jhung SH (2021) Adsorptive removal of pesticides from water with metal–organic framework-based materials. Chem. Eng. J. <https://doi.org/10.1016/j.cej.2021.129688>.

Navarathna CM, Dewage NB, Karunanayake AG, Farmer EL, Perez F, Hassan EB, Mlsna TE, Pittman CU (2019) Rhodamine B Adsorptive Removal and Photocatalytic Degradation on MIL-53-Fe MOF/Magnetic Magnetite/Biochar Composites. Journal of Inorganic and Organometallic Polymers and Materials. 10.1007/s10904-019-01322-w.

Nazir MA, Najam T, Bashir MS, Javed MS, Bashir MA, Imran M, Azhar U, Shah SSA, Rehman AU (2022) Kinetics, isothermal and mechanistic insight into the adsorption of eosin yellow and malachite green from water via tri-metallic layered double hydroxide nanosheets. Korean J. Chem. Eng. <https://doi.org/10.1007/s11814-021-0892-3>.

Nejadshafiee V, Islami MR (2020) Bioadsorbent from Magnetic Activated Carbon Hybrid for Removal of Dye and Pesticide. ChemistrySelect. <https://doi.org/10.1002/slct.202001801>.

Parsazadeh N, Yousefi F, Ghaedi M, Dashtian K, Borousan F (2018) Preparation and characterization of monoliths HKUST-1 MOF via straightforward conversion of Cu(OH)_2_-based monoliths and its application for wastewater treatment: artificial neural network and central composite design modeling†. New J. Chem. <https://doi.org/10.1039/C8NJ01067F>.

Sanz-Santos E, Álvarez-Torrellas S, Ceballos L, Larriba M, Águeda VI, García J (2021) Application of Sludge-Based Activated Carbons for the Effective Adsorption of Neonicotinoid Pesticides. Applied Sciences. <https://doi.org/10.3390/app11073087>.

Srikhaow A, Chaengsawang W, Kiatsiriroat T, Kajitvichyanukul P, Smith SM (2022) Adsorption Kinetics of Imidacloprid, Acetamiprid and Methomyl Pesticides in Aqueous Solution onto Eucalyptus Woodchip Derived Biochar. Minerals. <https://doi.org/10.3390/min12050528>.

Suo F, Liu X, Li C, Yuan M, Zhang B, Wang J, Ma Y, Lai Z, Ji M (2019) Mesoporous activated carbon from starch for superior rapid pesticides removal. Int. J. Biol. Macromol. <https://doi.org/10.1016/j.ijbiomac.2018.10.132>.

Thabet MS, Ismaiel AM (2016) Sol-Gel γ-Al_2_O_3_ Nanoparticles Assessment of the Removal of Eosin Yellow Using: Adsorption, Kinetic and Thermodynamic Parameters. J. encapsulation adsorpt. sci. <http://dx.doi.org/10.4236/jeas.2016.63007>.

Vukčević MM, Kalijadis AM, Vasiljević TM, Babić BM, Laušević ZV, Laušević MD (2015) Production of activated carbon derived from waste hemp (Cannabis sativa) fibers and its performance in pesticide adsorption. Microporous Mesoporous Mater. <https://doi.org/10.1016/j.micromeso.2015.05.012>.

Wang QY, Yang J, Dong X, Chen Y, Ye LH, Hu YH, Zheng H, Cao J (2020) Zirconium metal-organic framework assisted miniaturized solid phase extraction of phenylurea herbicides in natural products by ultra-high-performance liquid chromatography coupled with quadrupole time-of-flight mass spectrometry. J. Pharm. Biomed. Anal. <https://doi.org/10.1016/j.jpba.2019.113071>.

Wang X, Wang H, Lu M, Teng R, Du X (2017) Facile synthesis of phenyl-modified magnetic graphene/mesoporous silica with hierarchical bridge-pore structure for efficient adsorption of pesticides. Mater. Chem. Phys. <https://doi.org/10.1016/j.matchemphys.2016.12.017>.

Zhang M, Yu Z, Yu H (2020) Adsorption of Eosin Y, methyl orange and brilliant green from aqueous solution using ferroferric oxide/polypyrrole magnetic composite. Polym. Bull. <https://doi.org/10.1007/s00289-019-02792-1>.
